# Recent Advances in the Synthesis and Processing of Carbon Nanotubes and Carbon Nanocomposites for Energy Storage, Biomedical, and Environmental Applications

**DOI:** 10.1002/open.202500501

**Published:** 2026-04-20

**Authors:** Noor‐ul‐Huda Altaf, Muhammad Ayyaz, Yaning Zhang, Mudassar Shahzad, Ayesha Khan, Sanam, Wasqa Liaqat, Ambreen Ashar, Roshaan Malik, Zeeshan Ahmad Bhutta

**Affiliations:** ^1^ School of Energy Science and Engineering Harbin Institute of Technology Harbin China; ^2^ Department of Physics University of Agriculture Faisalabad Faisalabad Pakistan; ^3^ Department of Chemistry, Materials and Chemical Engineering “Giulio Natta” Politecnico di Milano Milan Italy; ^4^ Department of Physics Government College University Faisalabad Faisalabad Pakistan; ^5^ School of Chemistry and Materials Science Nanjing Normal University Nanjing P. R. China; ^6^ University of Management and Technology Lahore Lahore Pakistan; ^7^ Department of Chemistry University of Agriculture Faisalabad Faisalabad Pakistan; ^8^ The Langley Academy Langley Berkshire UK; ^9^ Laboratory of Biochemistry and Immunology College of Veterinary Medicine Chungbuk National University Cheongju Republic of Korea

**Keywords:** biomedical, carbon nanocomposites, carbon nanotubes, environment, fuel cells

## Abstract

Carbon nanotubes (CNTs) and carbonnanocomposites (CNCs) representsome of the most innovative materials in nanotechnology, finding applications in biomedical devices, aerospace, and environmental remediation. Recent developments have improved the synthesis of CNTs, including variants that are single walled (SWCNTs) and multiwalled (MWCNTs). This review discussed promising techniques like laser ablation, which improves the purity of CNT production, and new functionalization methods that maximize material performance. The mechanical strength, electrical conductivity, and thermal stability of CNTs and CNCs have all significantly improved because of these developments, making them extremely ideal for crucial applications, including improved structural materials in aircraft and cutting‐edge components in medicinal devices. This article provides a thorough analysis of these advancements, discuss great progress that has been made in the synthesis of CNTs and CNCs by developing techniques; like chemical vapor deposition (CVD), laser ablation, and plasma‐assisted techniques, and their effects on material properties. This review explores recent advances in CNTs and CNCs, highlighting key challenges and future research directions across various applications.

## Graphical abstract

This review summarizes recent advances in the synthesis and processing of carbon nanotubes and carbon nanocomposites, highlighting structure–property relationships and their applications in energy storage, biomedical systems, and environmental technologies, with emphasis on performance optimization and sustainable material design.

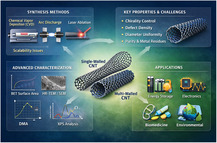

## 1 Introduction

**FIGURE 1:**
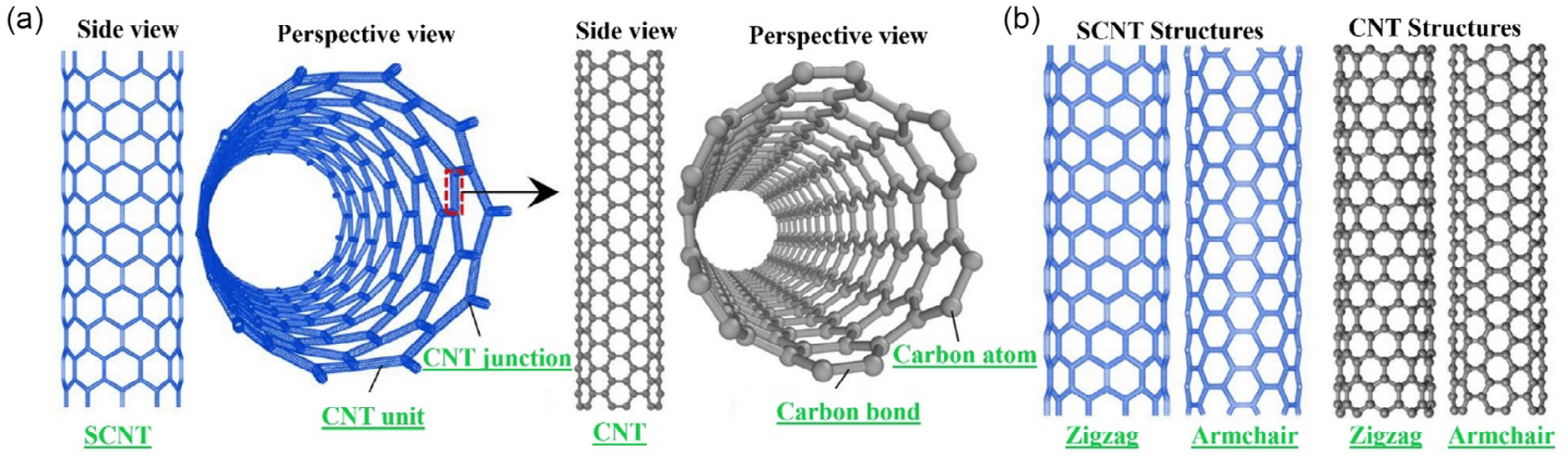
Shows the geometrical shapes of CNTs and SCNTs with zigzag and armchair chiralities, emphasizing their effects on electrical conductivity. (a) SCNT and CNT frameworks are illustrated. (b) The zigzag arrangement usually shows semiconducting behavior, whereas the armchair arrangement shows metallic characteristics. Adapted from [12].

Nanotechnology has become a revolutionary platform of scientific and technological innovation by allowing the accurate governing of materials at the Nanometer scale. Over the past few decades, nanoscience, nanotechnology, and nanocomposite engineering have experienced rapid development, resulting in major advances in a broad spectrum of industries, such as flexible electronics, energy storage, biotechnology, aerospace, automotive engineering, and environmental remediation [1, 2]. One of the reasons that has led to this growth is the fact that nanostructured materials can display size‐dependent physical, chemical, and biological properties not attainable in their bulk counterparts. 1D tubular nanostructures, including silicon nanotubes, silicon carbon nanotubes, and carbon nanotubes (CNTs), are particularly of interest because of their large surface areas, tunable electronic and chemical properties [3]. Simultaneously, nanoparticles (NPs) and nanocomposites (NCs) with very high ratios of surface‐to‐volume have shown high promise in electronics, medicine, and environmental cleanup technologies [4]. In this topography, carbon‐based nanomaterials such as CNTs, graphene, fullerenes, and their derivatives have a central role due to the allotropy, chemical versatility and stability of sp_2_‐hybridized networks of carbon [5]. In the last 20 years, CNTs and CNCs have led the nanomaterials research due to their outstanding mechanical strength, electrical conductivity, thermal stability, and chemical stability [6]. CNTs are structurally seamless cylindrical nanostructures that are made by rolling sheets of graphene with a diameter usually ranging between 0.7 and 50 nm and lengths ranging between micrometers to centimeters [7, 8]. Graphene and CNTs, in turn, are 2D crystalline sheets of sp^2^‐bonded carbon atoms, and CNTs inherit many of the excellent characteristics of graphene with additional curvature‐ and chirality‐related effects [9, 10]. Depending on the graphene layer, CNTs are broadly categorized as single‐walled (SWCNTs) and multiwalled CNTs (MWCNTs) with different electronic, optical, and mechanical properties due to their unique structural shapes [11]. Chirality of CNTs, often referred to as armchair, zigzag, or chiral, is a decisive factor in the electrical properties of CNTs, allowing them to act as either narrow‐bandgap semiconductors or metallic conductors (Figure 1a,b). This structure‐property relationship is a characteristic that forms the basis of the application of CNTs in nanoelectronics, sensors, energy devices, and biomedical platforms [13]. In addition, the delocalized *π*‐electrons are highly concentrated on the surfaces of CNTs, which enables the interfacial interactions with polymers, metals and ceramic matrices to be strong and CNTs are thus very effective fillers in multifunctional nanocomposites. Other carbon‐based hybrid systems like graphene‐ and reduced graphene oxide (rGO)‐based composites, which are typically used in conjunction with conductive polymers, like polyaniline (PANI), or conductive semiconductor quantum dots, have also increased the range of applications in sensing, optoelectronics, and energy conversion [14, 15].

Although these benefits exist, synthesis and processing plans have a strong influence on the large‐scale implementation of CNTs and CNCs. Conventional production techniques, including chemical vapor deposition (CVD), arc discharge, and laser ablation, offer varying degrees of control over the quantity, quality, and structural purity of CNTs. Although CVD is presently the most commonly used technique because of its relative scalability, the majority of the CNT synthesis pathways are energetically expensive, high‐temperature, and use expensive catalysts or facilities, which may limit economic and environmental sustainability [16, 17, 18]. This has led to the increased interest in emerging alternative synthesis and processing methods, which are economical, scalable, and environmentally friendly, without affecting material performance.

Here, the synthesis routes of CNT and CNC, processing approaches, and optimization of properties based on application urgently require critical and up‐to‐date evaluation. Although many studies mention individual progress, the body of literature is still disjointed, not always including direct comparisons between synthesis technologies and what this means to structure, properties, and application relationships. This review fills this gap by synthetically reviewing recent progress in the synthesis and processing of CNTs and CNCs, with an especially heavy focus on the effects of processing routes on the material properties and performance of the materials in energy storage, biomedical, and environmental applications. This review will deliver a clear framework that can be used to inform the underlying research and application of CNCs as it incorporates comparative analysis, highlights critical issues, and future research directions.

## 2 Carbon Nanotubes (CNTs)

CNTs are nanotubular structures rolled up on the sheets of graphene, consisting of sp_2_‐hybridized carbon with constituent atoms arranged in a hexagonal structure. The distinctive architecture gives them exceptional mechanical strength, high electrical conductivity and high thermal stability. These attributes give CNTs extreme usefulness in a wide range of applications, such as electronics, nanotechnology, materials science, and biomedical engineering. Generally, CNTs can be classified as SWCNTs, double‐walled (DWCNTs) and multiwalled (MWCNTs), which have different structure and function features. The critical influence on the electronic behavior of CNTs is chirality, which is characterized by the chiral indices (n, m), when it defines whether a nanotube is a metal or a semiconductor. This property that relies on the chirality is of special concern in nanoelectronics. Continued studies also focus on synthesis of parallel and chiral‐specific CNTs to improve further incorporation of CNTs into emerging technologies. Moreover, the development of mass production methods is broadening the useful application of CNTs, providing additional possibilities in high‐performance nanomaterials and devices of the next generation.

### 2.1 Single‐Walled Carbon Nanotubes (SWCNTs)

**FIGURE 2:**
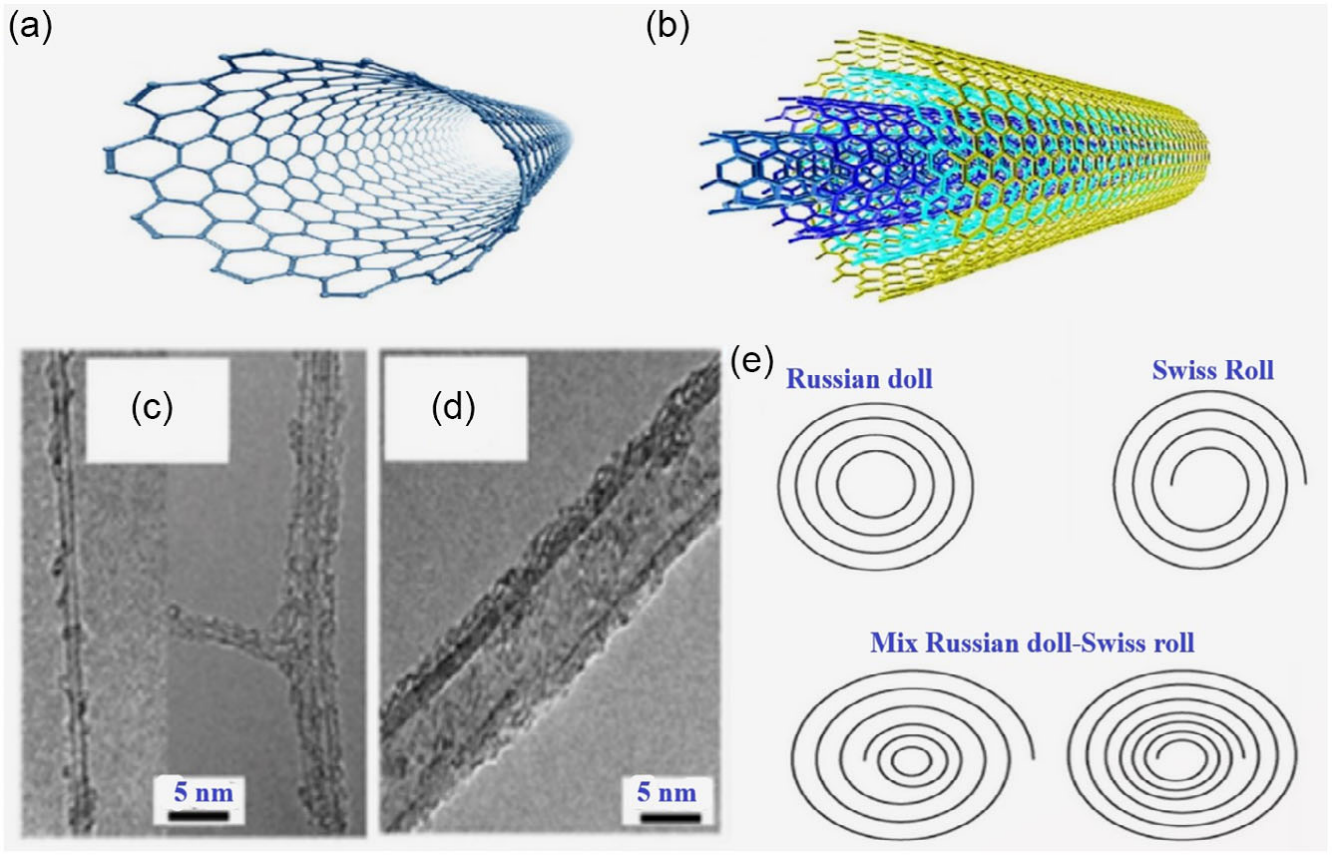
The structure of (a) SWCNT and (b) MWCNT, and HR‐TEM images of (c) SWCNT and (d) MWCNT. (e) MWCNT structural models of Russian doll, Swiss roll (Parchment model), and mix Russian doll–Swiss roll. Adapted from [22].

SWCNTs are cylindrically shaped and hollow and consist of a single layer of carbon atoms that are arranged in a tube shape. They have extraordinary characteristics, which depend on the chiral vector, which contributes to metallic or semiconducting character. The (n, m) indices in the chiral vector reflect how many unit vectors in the tube (along the tube axis) there are and how many around the circumference of the tube. This is the chiral vector of metallic SWCNTs in instances when n‐m is a multiple of 3. Their extreme high electrical conductivity and bandgap free nature make them ideal in applications with high performance in electronics devices and interconnects. The chiral vector of semiconducting SWCNTs (s‐SWNCTs) is that there are no multiple occurrences of n‐m that is a multiple of 3. The band structure of these CNTs can be changed by changing the tube diameter and chirality, thus utilizing these in such applications as transistors, sensors, and optoelectronic devices [19]. The primary goals of the research work have been the synthesis of SWCNTs with specific chirality and the investigation of their electrical properties. In 2022, Jiao et al. described the production of pure s‐SWCNTs by a floating catalyst CVD method. Due to the flexibility of their bandgap, the resultant SWCNTs were suggested [20]. Figure 2a [21] displays the schematic diagram of SWCNTs. SWCNT has a single graphene layer, while MWCNT has more than two graphene layers, according to the HR‐TEM images Figure 2c,d [22]. Each carbon atom in SWCNT is covalently bonded to three neighboring carbons via sp^2^ molecular orbitals and arranged in a hexagonal lattice. The fourth valence electron in the *p*_*z*_ orbital hybridizes with all the other *p*_*z*_ orbitals to form a delocalized *π*‐band. There are three different forms of SWCNT, such as armchair, chiral, and zigzag. On the other hand, MWCNT can be formed in three structural models, including a Russian doll, Parchment (Swiss roll), and a mix Russian doll–Swiss roll, as revealed in Figure 2e [23].

### 2.2 Multiwalled Carbon Nanotubes (MWCNTs)

MWCNTs are multilayered constructs formed by rolling layers of graphene into a cylindrical shape, as schematically represented in Figure 2b [21]. Although MWCNTs have inferior electrical conductivity compared to SWCNTs, their mechanical properties are exceptional due to the interactions between the layers, boasting high tensile strength, toughness, and application versatility. The Young's modulus of MWCNTs ranges from 270 to 950 GPa, with some measurements averaging 1.28 TPa, and their tensile strength can reach 63 GPa [24]. Additionally, MWCNTs prepared via arc discharge synthesis exhibited an average bending strength of 14 GPa, as reported by Wong et al. [25]. The use of MWCNTs as reinforcement materials in composites is quite common, as they possess the ability to enhance mechanical performance significantly. For example, Li et al. demonstrated that the introduction of MWCNTs into a polymeric matrix made the composite more durable and increased its tensile strength [14]. Zhao et al. also studied the effects of adding MWCNTs to the matrix and fiber of carbon–fiber‐reinforced composites, reaffirming the strengthening of short‐beam flexural and tensile strength at low loading levels [26]. MWCNTs tend to alter the performance of composites significantly; Prabhudass et al. [27] revealed that the incorporation of MWCNTs in natural fiber‐reinforced composites (NFRCs) increased glass transition temperature (*T*_g_) and storage modulus (E) by 41%. Numerous studies have been conducted on the impact of CNT reinforcement on the mechanical properties of metal‐based nanocomposites. The synthesized Cu/CNTs nanocomposites by Wei et al. proved this by escalating the glass transition temperature (*T*_g_) and storage modulus (*E*) by a factor of 41 on dipping MWCNTs in the NFRCs. The effects of adding CNTs to metal‐based nanocomposites on their mechanical properties have been of interest to many researchers. Wei et al. [27] synthesized the Cu/CNTs nanocomposites through the electroless deposition method. They demonstrated that modifying the Ni particles improved CNT dispersion, resulting in a 22% increase in tensile strength and a 41% increase in elastic modulus compared to pure Cu. In a similar vein, Simoes et al. [28] studied Al‐MWCNT nanocomposites. They found that the ideal composition, at 0.75 wt% MWCNTs, produced superior tensile strength and hardness within the Al matrix. Nouri et al. [29] reported that incorporating 2 vol% CNTs into Al composites led to a 36% and 13% enhancement in hardness and Young's modulus, respectively. A more significant improvement was observed in Ti‐based nanocomposites by Nezhad et al. [30], where Ti‐CNTs synthesized via hydrothermal and sol–gel methods exhibited a remarkable 191% increase in elastic modulus for Ti‐2 wt% CNTs compared to pure Ti. While the extent of mechanical property enhancement varies depending on the matrix material, synthesis method, and CNT content, all studies confirm that CNT reinforcement leads to significant improvements. The most pronounced increase in elastic modulus was observed in Ti‐based composites, whereas Cu and Al matrices showed notable but comparatively lower enhancements. The following Table 1 highlights the structural and mechanical differences between MWCNTs and SWCNTs, as well as their impact on composite materials.

**TABLE 1:** Provides a comparison of key mechanical properties between MWCNTs and SWCNTs.

Property	SWCNTs	MWCNTs	References
Young's modulus	∼1 TPa	270–950 GPa (up to 1.28 TPa)	[24]
Structure	Single graphene cylinder	Multiple concentric graphene layers	[21]
Fracture toughness	Moderate	High	^—^
Electrical conductivity	Higher due to single‐layer structure	Lower due to interlayer interactions	[21]
Tensile strength	∼50–150 GPa	Up to 63 GPa	[24]
Bending strength	Higher than MWCNTs	14 GPa (arc discharge method)	[25]
Thermal conductivity	∼3500 W/m K	∼3000 W/m K	[21]
Flexibility	Higher due to single‐wall nature	Lower than SWCNTs	[21]
Composite enhancement	Enhances electrical conductivity and mechanical properties but is less commonly used in bulk composites due to dispersion challenges	Improves tensile, flexural, and impact strength in polymers and metals	[14, 26, 27]

### 2.3 Functionalized Carbon Nanotubes (F‐CNTs)

Through chemical modification, different functional groups or NPs are added to the surface of functionalized CNTs (F‐CNTs). This modification increases CNTs’ potential applications across various industries and improves their compatibility with other materials. During functionalization, it is possible to drastically alter the chemical and physical properties of CNTs, making them more reactive and dispersible in matrices and solvents, and this opens new avenues for chemical alterations. One of the common types of functionalization is oxidation, which introduces oxygen‐containing functional groups to the surface of CNTs; such groups include hydroxyl (‐OH) and carboxyl (‐COOH) functional groups. The following is an example of the dispersion process:

#### 2.3.1 Oxidation Reaction

$$\mathrm{CNT}+O_{2}\;\to \;\mathrm{CNT}-\text{COOH}/-\mathrm{OH}$$

Upon dispersionto add carboxyl groups, CNTs become easier to disperse in polymers and are more compatible and easily dispersed in composites. To illustrate, the dispersion of carboxyl groups enhances the dispersion of CNTs in polymer matrices, leading to a 25% increase in the tensile strength of CNT composites [31]. This modification is beneficial for delivering drugs and CNCs, where improved solubility and functionalization are required to interact effectively with the molecule. Another method of functionalization is called Amination, which serves to add amino groups (‐NH_2_) to the surface of CNTs so that they become more soluble in various solvents. The process of dispersion, as shown below, enables CNTs to add carboxyl groups, making them easier to disperse in polymers and more compatible with composites. To illustrate, the oxidation of carboxyl groups enhances the dispersion of CNTs in polymer matrices, leading to a 25% increase in the tensile strength of CNCs

#### 2.3.2 Amination Reaction

Amination adds nitrogen‐containing functional groups (–NH_2_, –NH–) to the surface of CNTs. These groups improve interfacial bonding through hydrogen bonding and electrostatic interactions. This makes polymer matrices better at spreading and transferring loads without the severe structural damage that is often caused by oxidative treatments. The catalytic properties of CNTs may be improved by functionalizing CNTs with metallic NPs (platinum (Pt), silver (Ag) or gold (Au)) or nonmetallic NP (gallium arsenide (GaAs), carbon (C) or silicon (Si). An example of metallic NP deposition on CNTs is as follows:

$$\mathrm{CNT}+{\mathrm{NH}}_{3}\;\;\to \;\mathrm{CNT}-{\mathrm{NH}}_{2}\;$$

#### 2.3.3 Metal Deposition Reaction

CNTs find their best use in fuel cells and sensors, as well as catalytic reactions. The catalytic ability of CNTs is enhanced by the metal NPs that act as active sites of chemical reactions. Multiple methods have been employed to functionalize CNTs, improving their properties and expanding their applicability. As an illustration, Karimzadeh et al. employed carboxyl‐modified SWCNTs as nanocarriers. The high performance of drug loading and release properties of these functionalized SWCNTs gave hope to the research in the field of nanomedicine [32].

### 2.4 Aligned Carbon Nanotubes

CNTs have been keen on a particular mechanism of generating ordered nanotube arrays by applying the wrapping method. Several methods have been used to align them, including template‐assisted growth, electric field alignment, and CVD; possible applications of aligned CNTs, particularly field‐effect in 2021, Yang and coauthors demonstrated that controllable transistors with aligned SWCNT could be fabricated. Better charge carrier mobility was demonstrated by the aligned SWCNTs, making them suitable for use in high‐performance electronic devices [33]. The CNT transistors are at the nanoscale and operate at room temperature, unlike silicon. So, it is possible to substitute bigger size transistor with a smaller one to operate data with low resistance and higher speed. It provides high charge carrier mobility and exceptional electrostatic control owing to their thin structure. It has the capability to gain extremely small channel length which cause the quicker switching speeds and high current densities relative to conventional transistor which arebased on silicon. The carrier has high mobility and realizes ballistic transport in CNT field effect transistor due to a quasi‐1D structure. The hundreds of nanowires can be embedded as the conductive channel in a single CNT field effecttransistor, which enable it ideal current transport in low supply voltage. So, it provides a foundation basis for gaining ultralarge‐scale logic circuit at the nanoscale level [34] CNT show high charge carrier mobility as compared to conventional transistors made of silicon. The estimated increase in charge carrier mobility is up 10 times or more, which highly depends on a certain design and fabrication method. The high charge carrier mobility means faster execution and lower power consumption [35].

Due to their energy efficiency and scalability, aligned CNTs are a possible substitute for silicon in field‐effect transistors (FETs), as noted by Lin et al. [29]. With dimensions like 10 nm silicon node, this study shows how to fabricate aligned CNTs FETs with improved performance metrics. Moreover, use of a full‐contact structure reduces contact resistance, enabling the creation of nanotube FETs with a 55 nm gate pitch, surpassing the performance of 10 nm silicon metal–oxide‐semiconductor transistors [16].

### 2.5 Double‐Walled Carbon Nanotubes (DWCNTs)

As the name implies, two concentric nanotubes comprise DWCNTs. The chirality of the inner and outer tubes determines whether they are metallic or semiconducting. DWCNTs combine the mechanical properties of MWCNTs with some of the electrical characteristics of SWCNTs. Due to their potential for a wide range of applications, DWCNTs have drawn interest. Yang et al. report focused on the synthesis of DWCNTs and their application in high‐performance lithium‐ion batteries. The sample has been shown to have an increased capacity for storing lithium and cycle stability in DWCNT‐based anodes, which can be utilized in energy storage devices, as demonstrated through this study [33].

### 2.6 Chirality‐Specific Carbon Nanotubes

CNTs have been synthesized to possess a specific chirality, or chiral vectors (*n*, *m*), to obtain an accurate electrical property. Chirality control is key in applications where a specific electronic behavior is sought. There has been intense research on the potential use of chirality‐specific CNTs in nanoelectronic devices [36]. Zhang et al. were concerned with producing chiral‐specific SWCNTs. This study revealed that SWCNTs possessed top‐of‐the‐range transportation qualities of electrons that qualified them for use in high‐performance transistors. CNTs have been synthesized to maintain a specific chirality, or chiral vectors (*n*, *m*), to obtain accurate electrical properties. Chirality control is key in applications where a specific electronic behavior is sought. There has been intense research on the potential use of chirality‐specific CNTs in nanoelectronics devices [37]. To provide an in‐depth insight into the advancements made in the study concerning CNTs and CNCs, Table 2 presents a detailed comparison of notable research discoveries. This table presents research studies that have yielded significant results and innovations in various applications of different types of CNTs and CNCs.

**TABLE 2:** The key properties and applications of different types of CNTs are compared.

Types of CNTs	Superior findings and applications	References
SWCNTs	Synthesis of semiconducting SWCNTs with tunable bandgap for high‐performance transistors.	[20]
MWCNTs	Incorporation of MWCNTs into a polymer matrix for enhanced tensile strength and stiffness in composite materials	[15]
Functionalized CNTs	Carboxyl‐functionalized SWCNTs for improved drug delivery capabilities in nanomedicine.	[36]
Aligned CNTs	In terms of both performance and contact resistance, aligned nanotube FETs scaled to 10 nm outperform silicon transistors.	[38]
DWCNTs	Synthesis of DWCNTs for use in high‐performance lithium‐ion batteries with superior lithium storage capacity and cycle stability.	[33]
Chirality specific CNTs	Synthesis of chirality‐specific SWCNTs with chiralities, showcasing superior electron transport characteristics for high‐performance transistors	[37]
PCPP	Single wall carbon nanotube poly (cyclo‐*para*‐phenylene Used as a anode material in Li‐ion battery	[39]

## 3 Preparation Methods

CNTs and carbon nanocomposites (CNCs) can be efficiently synthesized using a variety of advanced techniques, encompassing chemical, physical, and electrochemical approaches. This versatility in production methods opens the door to innovative applications, enhancing the potential of these remarkable materials. There are two commonly employed processing methods: thermal synthesis processes, which consist of CVD and plasma‐enhanced CVD (PECVD), and plasma‐based fabrication methods, such as laser ablation and arc discharge. Among them, CVD is the most used method. During CVD, CNTs typically grow on a substrate due to the thermal decomposition of hydrocarbon gases in the presence of a catalyst [20]. The other notable application of arc discharge and laser ablation methods is the vaporization of carbon sources in various gas atmospheres [40]. Furthermore, recent advancements in the field have enabled new methods, such as solution‐phase processes and the chemical reduction of graphene oxide. The required properties of the CNTs, such as purity, scalability, and chirality, heavily influence the synthesis technique selection. Because of the combined efforts of scientists and engineers, the synthesis of CNTs is still being expanded, which explains the enormous potential of CNTs and CNCs in fields like materials science, nanotechnology, and electronics [41].

### 3.1 Chemical Vapor Deposition (CVD) Method

The properties of CNT are based on their chirality, dimensions and diameter, which depends on the different parameters involved in the synthesis technique, such as temperature, catalyst, concentration and carrier of gas. SWCNT attracted many researchers due to its outstanding structure and physical properties and is considered an essential substitute in the fabrication of nanoelectronics. Its unique properties depend on its particular chiral structure, which includes diameters, chiral angles, and hardness [42]. A chiral carbon refers to a certain arrangement of carbon atoms in its nanotube morphology. In this structure, the graphene sheet is rolled at an angle, creating a chirality (handedness). The chirality of CNT highly influences the electronic properties of CNTs. The chirality is controlled by the catalyst used in the CVD process. It basically depends on the growth conditions and catalyst used in the CVD process [43].

#### 3.1.1 Chirality Control Parameters in CVD Process

These are different factors that highly influence the chirality of CNT:

##### 3.1.1.1 Catalyst Selection

Different metal like Fe, Co, Ni, have distinguished tendencies to develop certain chiral nanotubes relative to their crystal structure and interaction with CNT [44].

##### 3.1.1.2 Temperature Effect on Carbon Nanotube

The temperature highly influences the chirality of CNT during its growth process. It basically affects the growth kinetics and the relative stability of various chiral structures. For instance, by using a different temperature range, a higher prevalence of certain CNT can be developed within a precursor. Higher temperature may lead to the development of a specific chiral structure as compared to lower temperature. For example, a four times increased growth rate has been observed when the temperature increases from 750°C to 950°C and the average diameter also improves from 30 to 130 nm [45].

##### 3.1.1.3 Morphology and Size of Catalyst

The dimension and morphology of NPs highly influence the diameter and chirality of the CNT, which can be controlled.

##### 3.1.1.4 Carbon Precursor Gas Selection

The selection of carbon precursor gas, such as, methane, acetylene, can influence the growth process of CNT and hence chirality.

##### 3.1.1.5 Why Chirality Is Important

It highly influences the electronic properties of CNTs. Such as the design of certain chirality showed the metallic, semiconducting or even superconducting properties which made them suitable for different sensors and electronic devices at the nanoscale. Precise control of chirality permits the fabrication of advanced material with certain functional properties [43].

**FIGURE 3:**
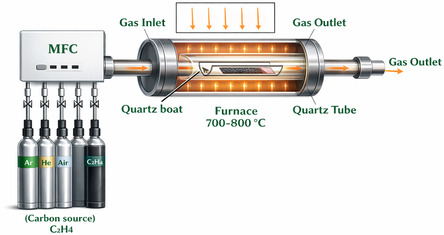
Schematic drawing of the CVD equipment employed in the synthesis of CNTs. Adapted from [50].

The CVD method has been employed for the synthesis of different carbon‐based materials. This method is renowned for its adaptability, scalability, and capacity to generate superior CNTs with regulated properties. One approach that appears to be economically feasible for producing CNTs on a wide scale is CVD. However, a major factor in deciding the end product's price is the cost of the carbon precursor [8]. In the CVD process, hydrocarbon gases are broken down in the presence of a metal catalyst, resulting in the formation of CNTs [38]. Different phases are involved in the CVD process. To provide nucleation sites for the formation of CNTs, a thin coating of metal catalyst, typically cobalt (Co), nickel (Ni), or iron (Fe), is first deposited onto a substrate during the catalyst preparation phase. The process is then started by introducing a hydrocarbon gas, such as methane (CH_4_) or ethylene (C_2_H_4_), into the CVD chamber. After that, the chamber is heated to temperatures over 600°C to produce carbon radicals from the precursor gas. These free radicals spread to the surface of the catalyst, where they are absorbed and grow vertically on the substrate to produce CNTs. Customization of CNT attributes, such as diameter, chirality, and structure, is possible through precise control of variables such as temperature, pressure, gas composition, and reaction time. Ultimately, following growth, the system is cooled, and the CNTs are gathered on the substrate. This substrate can be constructed of a variety of materials, including glass, stainless steel, CaCO_3_, Cu/Ti/Si, Ni, Cu, and SiO_2_. Alternatively, other investigations have used substrates like graphite and tungsten foil [46, 47, 48, 49]. According to Rather et al., MWCNTs were produced with porous MgO acting as a support and the trimetallic catalyst Co–Mo–V. The schematic drawing of the CVD equipment employed in the synthesis of CNTs is shown in Figure 3. The activation process takes place at 500°C in an alumina boat and involves the even distribution of the catalyst and support mixture. The activated sample is then placed in a horizontal quartz‐tube reactor to produce MWCNTs. Acetylene, containing hydrogen and carbon gas, is flown into the reactor. CVD has been used in the production of MWCNTs, and an interesting development has been that the surface area of MWCNTs increases as more metal is added to form a catalyst. This is directly linked to the rapid uptake of hydrogen [50].

Moreover, an examination of the potential role of mesoporous silica as a guide in the early stages of nanotube growth has been carried out. The importance of iron‐based compounds as catalysts, particularly in enhancing the solubility of carbon, was emphasized by Llamas et al. in the context of harnessing large‐scale production of graphene‐like nanomaterials. They also achieved the category of crystalline multilayer graphene flakes (MLGFs) through a two‐step catalytic CVD (CCVD) process using an alpha‐Fe catalyst and reduced nanostructured hematite. The first stage of the work involved the fabrication of nitrogen‐doped MWCNTs using a successful process, followed by Raman spectroscopy to confirm the presence of MLGFs. Thermogravimetric data demonstrated the possibility of using other types of carbon as the base for forming graphene; the maximum oxidation rate of the test was 643.5°C, with an estimated additional density of graphene growth on the oriented (110) surface of alpha Fe [51].

Viswanathan et al. devoted their attention to the synthesis of CNTs through the CVD procedure and the milling of CNTs and NC metal ZnO balls. They determined the structural morphology of the materials and used 1‐tetradecyl‐3‐methylimidazolium chloride, a cationic ionic liquid (IL), to enhance the stability of the nanocomposites. ZnO surface‐active sites spanning the hydroxyl and C groups of CNT‐ZnO NPs were identified by FTIR spectroscopy and confirmed by FESEM EDAX investigations. UV–vis spectroscopy measured the wavelengths of absorption and transmittance, and the *I*–*V* property suggested possible uses for their synthesized materials in gas sensors and piezoelectric devices, as well as in stress sensors [52]. Although, Yang et al.'s 2023 produced vertically aligned CNTs (VACNTs) in situ on catalyst‐treated CF surfaces, forming a 3D fiber‐net structure around the CF by CVD process. Additionally, resin precoating (RPC) guided epoxy resin into nanoscale spaces, eliminating void defects. The treated CFRP composites exhibited a 27.1% improvement in flexural strength, with altered failure modes indicating enhanced toughness and reduced delamination. Overall, the joint treatments of VACNT growth via CVD and RPC showed promise for manufacturing stronger CFRP composites suitable for aerospace applications [53].

The problem of increasing electric‐ionic conductivity and reducing volume change in Si‐based anodes was employed by Kim et al. in 2023. To produce spherical granules, they combined spray drying with a process known as CCVD. These granules contained silicon NPs (Si‐NPs) coated with nitrogen‐doped CNTs (Si‐NCNTs). Compared to Si‐NPs and commercial CNCs (Si‐CNTs) prepared by spray drying only, the Si‐NCNTs also showed better rate capability and initial discharge and charge capacities of 2457 and 1820 mA h g^−1^, respectively. They were found to display 57% retention after 200 cycles. It was also found that the Si‐NCNTs Li^+^ ion‐diffusion coefficient (DLi^+^) was almost threefold greater than those of the Si‐CNTs. Such inconsistency can be attributed to the ability of NCNTs to facilitate Li^+^ ion transport and buffer electrical conductivity among Si‐NPs [54]. The mechanical properties of vertically aligned carbon nanowalls (C‐NWs), a type of radial injection plasma enhanced chemical vapor deposition (RI‐PECVD)‐grown vertical sheets of graphene, were reviewed by Ghodkhe and colleagues in 2022. These CNWs have a superior modulus E approximate value at 28 GPa as well as a compressive strength of 50 MPa than that of other graphene‐based materials. Their elastoplastic characteristic, essential to the reliability and performance of the equipment, has been shown by high‐definition microscopy and provided an insight into the process of transformation in stress‐induced deformation [55]. Lin et al. in situ synthesized CNTs on powdered Ti‐6Al‐4V (TC_4_) alloy by applying PECVD. This technique allows the superior morphology and dispersion of CNTs compared with traditional CVD methods. Morphological and graphitic studies conducted on CNT growth behavior under varied conditions of PECVD, involving XRD, SEM, TEM, and Raman spectroscopy. The optimal conditions were found to be 600°C and a 30 min holding time, along with 50 W of plasma power, to produce a suitable quantity and quality of CNT on TC_4_ powders [56]. The aim of Table 3 is to summarize the methods and the outcomes of CNTs and CNCs by using different substrates:

**TABLE 3:** Synthesis overview of CNTs and CNCs on various substrates on various applications.

Synthesis method	Substrates/techniques used	Main findings	References
Two‐step catalytic chemical vapor deposition	Reduced nanostructured hematite, *α*‐Fe catalyst	Produced crystalline multilayer graphene flakes (MLGFs) and nitrogen‐doped MWCNTs.	[51]
CVD and Ball Milling	CVD for CNT synthesis, ball milling for CNT‐ZnO nanocomposites	Enhanced stability of CNT‐ZnO nanocomposites using cationic ion liquid (IL). Identified ZnO surface‐active sites.	[52]
CVD	In situ growth of vertically aligned carbon nanotubes (VACNTs) on CF surfaces	Significant improvement in flexural strength and toughness of treated CFRP composites, suitable for aerospace applications.	[53]
Catalytic chemical vapor deposition (CCVD) and dpray Drying	CCVD and spray drying for Si‐NP synthesis and coating with NCNTs	Si‐NCNTs exhibited enhanced rate capability and improved Li+ ion‐diffusion coefficient, suitable for battery applications.	[54]
Radial injection plasma enhanced chemical vapor deposition (RI‐PECVD)	Vertically aligned graphene sheets (carbon nanowalls)	CNWs demonstrated superior compressive strength and modulus values compared to other graphene‐based materials, suitable for device applications.	[55]
Plasma‐enhanced chemical vapor deposition (PECVD)	In situ synthesis of CNTs on Ti–6Al–4V alloy powder	Identified optimal conditions for satisfactory CNT quantity and quality on TC_4_ powders, suitable for various applications.	[56]
Arc discharge	Carbon nanomaterial synthesis using arc discharge	Systematically optimized process parameters and evaluated dye adsorption capacity, indicating potential for environmental remediation applications.	[57]

### 3.2 Laser Ablation Method

**FIGURE 4:**
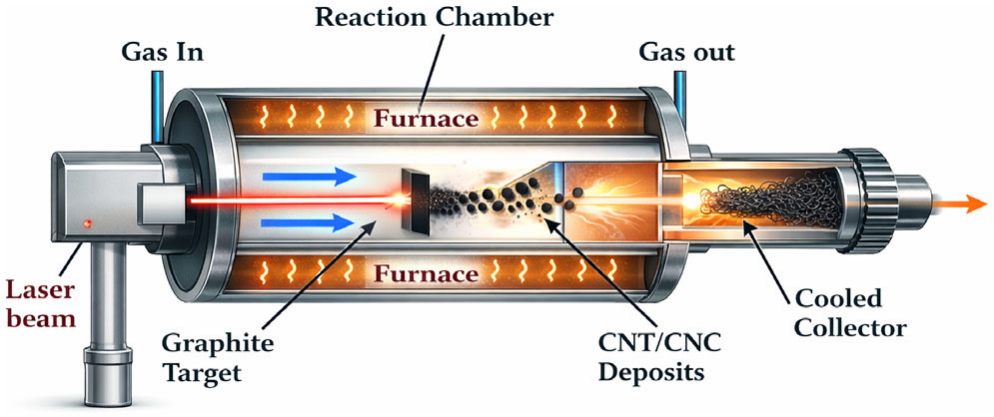
Illustration of laser ablation method for production of CNTs and CNCs. Adapted from [58].

SWCNTs of superior quality with regulated characteristics may be produced using a well‐known method for CNTs synthesis called the laser ablation method. Figure 4 shows the comparative contributions of scientists in the synthesis and processing of CNTs and CNCs by laser ablation method. Following is a description of the laser ablation method: A carbon‐containing target material, usually graphite or a carbon‐containing metal catalyst, is the focus of a high‐power laser. The target material vaporizes because of the laser's energy‐producing very high temperatures. The target material's carbon is vaporized by the laser's extreme heat. As it cools, the carbon vapor condenses to create CNTs. The energy, wavelength, duration, and target material composition of the laser are precisely regulated to customize the diameter, chirality, and structure of the synthesized CNTs. After the laser ablation process, the CNTs are collected as a product, which may require additional purification. Both MWCNTs and SWCNTs are synthesized using this technique. To grow SWCNTs with a diameter typically ranging from 1.0 to 1.6 nm, metal particles, often catalysts, are introduced into the graphite.

Several factors contribute to the properties of CNTs produced via the laser ablation method. These include the structure and chemical composition of the target material, ambient temperature, laser characteristics such as peak power, laser type (continuous or pulsed), oscillation wavelength, repetition rate, pressure of the buffer gas, chamber pressure, and the distance between the target and substrate [21, 59]. Notable literature and research work conducted by Baroot et al., investigating different uses of laser synthesis techniques for CNTs and their CNCs. The first study demonstrated the effectiveness of SnO_2_/CNT nanocomposites in photocatalysis for the degradation of MB dye by synthesizing and characterizing them using pulsed laser ablation. The structure and content of the NCs were validated by characterization methods such as TEM, SEM, XRD, and UV–visible spectroscopy; their photocatalytic activity under UV irradiation showed effective MB dye degradation according to pseudo‐first‐order kinetics. The study highlighted how these NCs strong dye degradation capabilities could be used to remediate wastewater [60]. In the second investigation, the production of AuNP and Au/CNT NCs for cancer therapy was examined using laser synthesis. Their bio‐compatibility as a major good was also established to be low when in contact with normal embryonic kidney cells without any toxicity, and when exposed to colorectal carcinoma and cervical cancer cells, it was identified as highly toxic [60]. Finally, the role of the pulse repetition rate in defining the chemical composition and mechanical characteristics of the laser‐treated material was investigated in the work by Musse and Lee on CNCs bipolar plates for proton exchange membrane fuel cells (PEMFCs). These results are of great value for the practical implementation of manufacturing laser‐processed CNT composites in a wide range of contexts, as a finding reveals that controlling pulse repetition rates is crucial in ensuring that there are no negative consequences [61].

Scientists have made considerable contributions to the development of CNTs using the laser ablation technique. Diversities in the properties of the synthesized CNTs have been caused by variations in target materials, catalysts and laser settings. There has been some work to maximize the production of SWCNTs with certain properties including controlled chirality and high purity, and some to test possible applications in composites and nanoelectronics. The different research goals and approaches utilized have added to the knowledge and support of SWCNTs. Among the major contributions includes synthesis of high‐quality SWCNTs that has been accomplished through laser ablation method. The researchers have employed this method in trying to investigate the numerous characteristics and potential applications of SWCNTs to develop the CNTs science and technology. The benefit of this process is that high yield CNTs are obtained compared to lower yield but with a lot of metallic contaminants. This drop in contaminants is explained by metallic catalysts’ to evaporate from the tube's end once it is closed. The final product made using the laser ablation process typically contains 70%–90% CNTs by weight. Compared to arc discharge, laser ablation offers greater control over the diameter of the CNTs by varying the reaction temperature. Additionally, SWNTs with laser ablation have fewer graphitic or amorphous carbon impurities [21, 62]. However, it should be noted that methods such as CVD or arc discharge are typically less expensive than laser ablation. This is mainly because this method depends on high‐purity graphite rods and high‐power lasers, which raises overall costs. The fact that the CNTs produced by laser ablation are frequently not uniformly straight and may branch is another disadvantage. Furthermore, compared to the arc discharge method, the CNT yield from laser ablation is usually lower [21]. Two novel methods are used to produce SWNTs on a large scale: the ultrafast pulse generated by a free electron laser (FEL) and the continuous wave laser powder approach. Despite their scalability, these techniques are rather costly because they require strong lasers and a large amount of energy input [21, 62, 63].

### 3.3 Flame Synthesis

**FIGURE 5:**
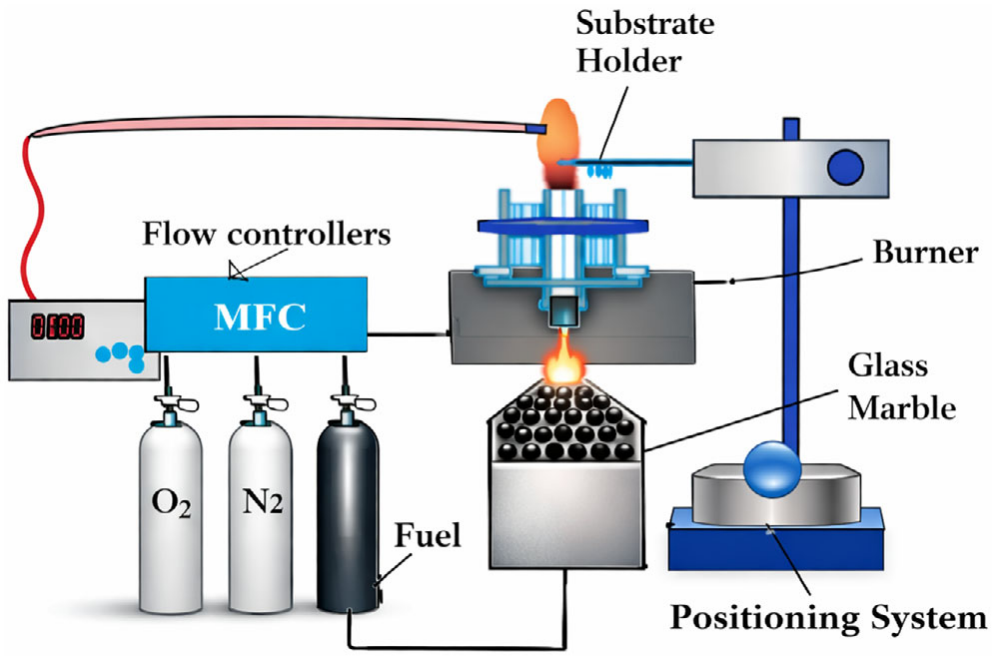
Schematics of flame chamber to produce CNTs. Adapted from [64].

Flame synthesis, a technique that involves the regulated oxidation of hydrocarbon fuels at high temperatures, can be used to generate CNTs. The simplicity, scalability, and potential for cost‐effective production of CNTs and other carbon materials by flame chamber, as illustrated in Figure 5, have attracted a lot of attention to this technique. Fuel combustion is the first step in the process, which produces a high‐temperature flame by burning hydrocarbon fuels like ethylene or methane in the presence of oxygen. In combustion, carbon NP nuclei are formed that become the nuclei on which CNT grow like growth habitats. During CNT forming, the temperature of flame lowers, and the carbon atoms combine into the growing nanotube metal structure and CNT creation occurs within the surfaces of these particles. The resulting CNTs are then harvested, usually on a surface or other deposition method, and thoroughly characterized to determine their structural, morphological as well as their physicochemical characteristics. The scalable and efficient flame synthesis method allows the production of CNTs with tunable properties, and it thus holds considerable potential to diverse industrial uses [64]. Some crucial factors for CNT growth in flame synthesis are as follows: The flame temperature has a major impact on the type and quality of CNTs produced. The size of the carbon particles produced by the flame affects the CNTs’ diameter. Sometimes, catalysts can be added to promote the growth of CNTs. The growth and development of CNTs are influenced by the fuel‐to‐oxygen ratio as well as other elements of the flame gas mixture. Using flame synthesis to generate CNTs has several significant advantages. This technique is highly scalable and cost‐effective approach that can be employed to produce CNTs on a large scale [65]. It also offers an edge over more complicated processes, like arc discharge or CVD, because of its simple process and equipment [66]. Furthermore, the high rate of production of CNTs, which lies between a few seconds to minutes, makes it highly well‐placed for high‐throughput applications [17]. The flame synthesis process is repeatable, which increases production efficiency and minimizes material usage, consequently lowering manufacturing costs [67]. Nevertheless, there are some key limitations associated with flame method synthesis, as the key properties of CNTs (such as diameter and chirality) can only be moderately controlled, thus preventing their further use in instances where their properties must be strictly defined [68]. Additionally, the length of flame‐generated CNTs is shorter, making them unsuitable for applications that require long CNTs [69]. Moreover, CNTs grown through the flame process are, in most cases, more contaminated by the presence of amorphous carbon and structural flaws, leading to impurities and inferior quality compared to those generated using more refined methods, such as CVD [70]. The structural configuration (chirality) of CNTs is hard to control, and often, a mixture of various types of CNT is obtained [71]. The high‐temperature flame in this approach may be energy‐intensive and not environmentally friendly [72]. This method involves a high‐temperature high‐temperature flame, which can be energy‐consuming energy‐consuming and ecologically unfriendly.

The MWCNTs were synthesized by Mahmoud et al. through the liquefied petroleum gas (LPG) in a self‐designed reactor by the approach of flame fragment deposition (FFD). Purification of the synthesis postsynthetic purification by ultrasonic treatment was performed with hydrogen peroxide (H_2_O_2_) and acetone to eliminate substances of combustion and polycyclic aromatic hydrocarbons (PAHs). The XRD, TGA, Raman spectroscopy, SEM, EDS, and TEM analyses confirmed the production of MWCNTs with a purity of more than 65%, having diameters of 31.26 and 78.00 nm [73].

To improve knowledge about the growth dynamics of CNTs in a flame, Ibrahim et al. compared CNT synthesis via premixed flames with methane diffusion. They discovered that the flame arrangement had a significant influence on the morphology and crystallinity of CNTs, as there was a difference in the distribution of temperature. Whereas the flames burned premixed, the view of the flame revealed a constant distribution of temperature across the radials, with an observable vertical difference. The diffusion flames showed three discrete regions of temperature development along the height of the burner (HAB). The changes in flame temperatures resulted in different CNT properties, including a 66% variation in crystallinity and a 44% variation in diameter. The article also emphasizes the use of flame arrangement in regulating the characteristics of CNTs to produce them in large quantities, suitable for applications such as energy storage and NCs, among others. Overall, these results demonstrate the importance of flame‐based synthesis techniques in modifying CNT properties for specific applications [74]. Moreover, the working environment of flame synthesis is hot, and the flame is open, requiring encapsulation. Unlike CVD, Hsu et al. reported in situ nanostructured carbon material synthesis with alcohol flames of Bunsen lamp burner (catalytic Ni substrate). Four liquid fuels were used: ethanol, n‐butanol, and their aqueous ammonia mixes. The effects of additive ammonia on flame temperature and the maximum temperature's axial position were demonstrated by the results. While ethanol with ammonia only created CNTs, n‐butanol with 10%, 20%, and 30% ammonia produced both CNTs and multi shell carbon nanostructures. The soot particles showed concentrically stacked carbon layers (diameter: ˜45 ± 5 nm), and the root growth of CNTs was different from the top growth in that, it did not involve encapsulated Ni catalytic particles at closed tips. The production of graphene took place in n‐butanol flames, demonstrating the differences in fuel types. High crystallization was observed in multi shell carbons and CNTs (IG/ID > 1) by Raman analysis. This work presents an easy‐to‐use and resource‐efficient method of flame synthesis using an alcohol Bunsen burner, which provides a quick and efficient substitute for traditional techniques [75]. The problem of creating functional fillers in polymer matrices for flame retardancy and electromagnetic interference (EMI) shielding in electronic devices is discussed by Chang et al. By using freeze‐drying, they establish a porous CNT bridged MXene (MXene/CNT) skeleton, guaranteeing barrier integrity and electrical continuity. In comparison to pure EP, the resulting MXene/CNT supported epoxy (EP) composite achieves improved flame retardancy and effective EM shielding (34.7 dB). Its low MXene/CNT content (1.7 wt%) also results in reduced heat release, smoke, CO, and CO_2_ production rates. This strategy presents a viable routefor the production of polymer composites [76].

### 3.4 Arc Discharge Method for Carbon Nanotube Synthesis

**FIGURE 6:**
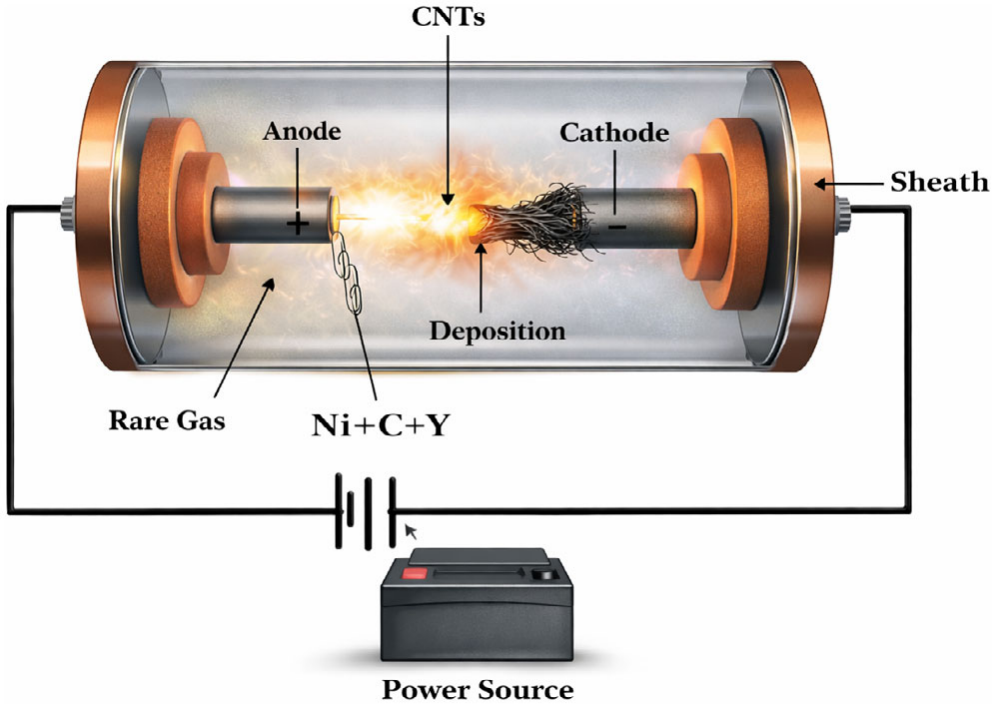
Schematic of arc discharge for CNT production. Adapted from [77].

The electrical arc discharge method is one of the first and oldest methods for producing CNTs which requires two graphite electrodes and an inert gas environment (such as helium) to create a high‐current electric discharge. Despite having been important in the early discovery of CNTs, it is still relevant in the production of MWCNTs. This study explores the arc discharge approach, summarizing important findings and different studies by scientists to use this approach to create CNTs and Figure 6 shows the representation of the arc discharge method to produce CNTs. Usually, an inert gas‐filled chamber contains graphite rods positioned closely together to serve as the anode and cathode. The anode is where carbon is vaporized by intense heat to generate carbon vapor, which is where CNTs growth mostly takes place [77]. This vapor condenses to generate CNTs, which are typically removed as soot, on the cathode surface. The structure of MWCNTs made using this approach is dependent on the synthesis parameters, such as the composition of the catalyst and the gas environment. While MWNTs were typically produced without catalysts, SWNTs were frequently produced using transition metal catalysts in the arc discharge method, which was a key tool in early CNTs research. In arc discharge setups for SWCNT synthesis, composite anodes made of graphite and different metals, such as Fe, Ni, Pd, Co, or their mixtures, are commonly used [78]. The process yield is highly influenced by the selection of the metal catalyst. A steady current density and anode consumption rate are essential for maintaining high efficiency, achievable only by keeping the spacing between the electrodes constant. Arc discharge is a technique that yields a mixture of components, necessitating the separation of nanotubes from the soot and catalytic metals present in the crude product. Both SWCNT and MWCNT can be synthesize during this method, as described by different authors [79]. Mehdi et al. compared the field emission performance of AD‐synthesized MWCNTs and CVD‐synthesized MWCNTs had lower turn‐on and threshold voltages repeated cycles of emission tests, higher crystallinity, and overall better performance than CVD‐MWCNTs. Additionally, lifespan tests of AD‐MWCNTs revealed increased long‐term stability, indicating their potential use as high‐performance field emitters [80]. The production of manganese NPs (Mn‐NPs) through thermal plasma arc discharge (TPAD) was studied by Kumaresea et al. Mixed manganese oxynitride (Mn_*x*_ON) was synthesized in different gas conditions and plasma power conditions, respectively, in addition to the various phases of manganese oxide (Mn_3_O_4_) and manganese nitride (Mn_3_O_4_). These Mn_3_O_4_ NPs, with their high specific capacitance and cycling durability, make them a highly efficient material of an electrode to be used in supercapacitors [81]. Wang et al. also employed rotational mixing and spark plasma sintering (SPS) to produce SWCNT‐reinforced Cu matrix composites. SWCNTs substantially improved the composites’ mechanical properties, including their ultimate tensile strength and elongation rate, while maintaining their superior electrical conductivity. Nanoscale Ni particles developed during the SWCNT generation process facilitated the connection between the SWCNTs and the Cu matrix, thereby increasing the composites’ ductility and altering their fracture mode. These findings highlight the adaptability of Cu composites reinforced with SWCNT [82]. Mohajer et al. 2023 pointed out the broad spectrum of MXenes and their composites in co‐catalysis, sensors, energy storage, electronics, and tissue engineering. Among this cluster, the MXene‐CNT composites have advantages due to their enhanced electrical and mechanical properties, and environmental stability. Through these advancements, it is now easier to develop highly advanced diagnostics, medical technologies, and environmental monitoring solutions. Superior physical properties, high electrical and thermal conductivity, and unique structural characteristics are all attributed to the incorporation of CNTs into MXenes. Even though electronics, sensing, and catalysis have advanced significantly, more research is required for biomedical and diagnostic applications, especially in the areas of biocompatibility, toxicity, and clinical translation [83]. Madhurima et al. used the arc discharge technique to synthesize carbon nanomaterials, optimizing process parameters like gas pressure and arcing voltage to increase yield and quality. Furthermore, the measurability of these carbon nanomaterials was evaluated using their ability to adsorb dyes, specifically Methyl Orange (MO) and Rhodamine B (RhB) dyes. According to the study, CNTs performed somewhat well in removing dye (16% for MO and 60% for RhB), but carbon soot, with its large specific surface area, showed exceptional efficacy (53% for MO and 82% for RhB). These results demonstrate the potential of carbon NPs for use in environmental remediation and point to directions for future study in functionalization methods to improve overall effectiveness. Furthermore, the difficulty of regulating the chirality of the CNTs and their often‐smaller size of less than 1 mm are constraints on this approach [57]. An overview of the research on the synthesis techniques and results of CNTs in a Table 4.

**TABLE 4:** Conditional synthesis and processing of various CNTs for different applications.

Type of CNTs and composites	Findings	Applications	References
SWCNTs and MWCNTs	Metal catalyst selection significantly influences process yield. Maintaining a steady current density and anode consumption rate is crucial for high efficiency. The arc discharge method produces a mixture of components, requiring the separation of nanotubes from soot and catalytic metals.	Various applications, including nanoelectronics, sensors, and reinforcement in composites.	[79]
MWCNTs	Arc discharge‐synthesized MWCNTs show lower threshold and turn‐on voltages, higher crystallinity, and improved long‐term stability compared to CVD‐synthesized MWCNTs.	High‐performance field emitters, nanoelectronics, sensors.	[80]
Manganese‐based nanoparticles	The thermal plasma arc discharge method produces manganese‐based nanoparticles with specific phases like Mn_3_O_4_, Mn_*x*_ON, and Mn_3_N_2_, showing exceptional specific capacitance and cycle stability, making Mn_3_N_2_ a promising electrode material for supercapacitors.	Supercapacitors	[81]
SWCNTs	SWCNT‐reinforced Cu matrix composites exhibit enhanced mechanical properties such as ultimate tensile strength and elongation rate. The connection between SWCNTs and Cu matrix, facilitated by Nanometer Ni particles, improves ductility and fracture mode.	Reinforcement in Cu matrix composites, thermal management materials, conductive additives.	[82]
MXene‐CNT composites	MXene‐CNT composites offer improved mechanical, electrical, and environmental stability, leading to innovative applications in electronics, cocatalysis, sensors, batteries, and tissue engineering. MXenes combined with CNTs provide unique properties.	Electronics, cocatalysis, sensors, batteries, tissue engineering.	[83]
CNTs and carbon soot	Optimized process parameters in arc discharge synthesis of carbon nanomaterials improve yield and quality. CNTs and carbon soot exhibit potential for environmental remediation, with carbon soot showing exceptional efficacy in dye adsorption.	Environmental remediation, functionalization methods development.	[57]

Amorphous C‐NPs, amorphous nanofibers, and carbon soot‐containing fullerene molecules are also produced by this technique, which lowers the total yield. Still, this approach is thought to be less costly than laser ablation, even with its reduced yield [84]. To increase productivity and agricultural mechanization levels, Li et al. investigates the use of CNTs in combination with cast steel for small agricultural machinery castings. The convergence of many carbon atoms gives CNTs their distinct features. At the nanoscale, they are characterized by a coaxial hollow tube structure. The study begins with a summary of the properties, structure, and preparation techniques of CNTs before diving into a qualitative examination of China's development of agricultural equipment. Cast steel and CNCs materials are made through experimental research, and the threshold model is used to analyze the data and determine the degree of mechanization. The results indicate that adding CNTs to cast steel improves the productivity of castings used in small agricultural machinery by enhancing mechanization. Furthermore, the research would also highlight the significant threshold effects, which are 5.641, 3.645, and 2.756, representing large, medium‐sized, and small machinery, respectively, in a grain‐sown area percentage. These threshold effects can provide major inflection points to promote agricultural mechanization [46].

### 3.5 Plasma‐Enhanced Chemical Vapor Deposition (PECVD)

A modification of CVD involves a process called PECVD, where plasma serves to boost the development of CNTs. The precursor gases are excited by plasma enhancement, which aids in CNT growth and forms carbon radicals. The most significant advantage of PECVD is that it enables the reduction of synthesis temperatures to lower levels than those used in conventional CVD processes. PECVD yields better control over the CNT growth process and is particularly useful in applications such as sensors and field emitters [85]. The species in the PECVD are easily dissociated in plasma, allowing for the growth of CNTs at relatively lower temperatures. These regulations lead researchers to select PECVD as a preferred technique for tailoring CNT characteristics to meet specific application requirements [86]. Growth rates are two orders of magnitude higher using PECVD. Both SWNTs and MWNTs have been grown on PECVD systems [87]. PECVD includes both direct and remote configurations for synthesizing CNTs. Direct PECVD is effective for growing MWNTs and selected SWNTs, while remote PECVD also supports both types. In a typical SWNT synthesis via direct PECVD, researchers used a CH_4_/H_2_ gas mixture at 500 mTorr, a plasma power of 900 W, substrate temperatures ranging from 550°C, to 850°C, and an external magnetic field [88]. Plasma plays a key role in SWNT formation, with energy modes shifting from low (60 W) to high (100 W) as power increases in an atmospheric pressure RF discharge reactor, thereby altering growth dynamics [89]. PECVD works using glow discharge because the high‐frequency voltage is applied between electrodes. Uniform growth of the CNT films is made possible by depositing a catalytic metal (e.g., Fe, Ni, Co) on substrates such as Si, SiO_2_, or glass using short‐distance CVD or sputtering techniques [88]. PECVD, as well as hot‐wire CVD (HWCVD), represents a hybrid approach that combines plasma‐based growth and CVD synthesis. In contrast, methods such as arc discharge, laser ablation, and solar furnace, PECVD relies on feedstock gases like methane (CH_4_) and carbon monoxide (CO) as carbon sources, eliminating the need for a solid graphite source. Argon‐assisted plasma is employed to break down the feedstock gases into reactive carbon species (C_*x*_H_*y*_), such as C_2_ and CH, facilitating growth at lower temperatures and pressures [90]. The advantage of using the PECVD method is the built‐in electric field which is present in a plasma sheath while adjusting the developing CNTs along the electric field lines.

### 3.6 Hydrothermal Method

There are several ways to create CNTs, one of which is hydrothermal technology, which uses water at a high temperature and pressure to help carbonaceous materials develop CNTs. It generally involves the following key steps: running on deionized water in a high‐pressure reactor vessel that tolerates severe conditions, using transition metal catalysts, such as iron, nickel, or cobalt NPs, and selecting a carbon source, typically glucose or sucrose. This process includes the dissolution of carbonaceous precursors in deionized water, mixing transition metal salts with reducing agents to create NPs, and subsequently adding the catalyst NPs to ensure uniform dispersal [91].

Production techniques for CNTs can be produced in many ways, but all involve attaching a catalyst to the start of a CNT, where one can attach the desired starting end. Hydrothermal methods use water at high pressure and temperature to promote the growth of CNTs on carbonaceous materials. The most common procedural steps are the following ones: the process can be done with a transition metal catalyst, such as iron, nickel, or cobalt NPs, a source of carbon, such as glucose or sucrose, and deionized water that is used in a reaction vessel of high pressure that can sustain such extreme conditions. The deionized water is added to the carbonaceous precursor. The transition metal salts and reducing agents are then mixed to form NPs, and the catalyst is thoroughly added to the NPs to create a uniform dispersion [92].

Hydrothermal synthesis has also been considered due to its scalability and the ability to create CNTs in a controlled manner. It is less expensive, experimentally simple, and employs environmentally friendly solvents such as water. Usually, CNTs are synthesized in high‐temperature and high‐pressure environments with the help of a transition metal catalyst such as iron. Chen et al. were able to synthesize MWCNTs using iron and glucose as catalysts and carbon sources, respectively, reaching a very high level of structural uniformity that could well be used in several applications [93]. To achieve better control over CNT shape and diameter, Tian et al. investigated the hydrothermal synthesis of nickel‐based catalysts using sucrose as a carbon source. Their study demonstrates the potential of the method to produce high‐quality, sustainable CNTs [94]. There should also be safety precautions when handling potentially hazardous materials, such as metal catalysts and high‐temperature, high‐pressure reactions. Sufficient lab materials and protection are required. All alternative CNTs manufacturing processes have their advantages and may be adapted to any industrial or research need. Researchers have investigated ways to produce CNTs with controlled properties and purity, expanding the potential applications of these incredible nanomaterials in fields like composites, electronics, and drug delivery. The expected applications and necessary characteristics of CNTs are summarized in Table 5. which affects the choice of synthesis method.

**TABLE 5:** Comparative literature analysis with key findings of CNTs and CNCs correlated with synthesis methods and possible applications.

Type of CNTs and composites	Methodology	Key findings	Applications	References
MWCNTs	Arc discharge	Arc discharge produces MWCNTs with an interlayer spacing of 0.34 nm and ideal crystallinity. Amorphous carbon nanoparticles, nanofibers, and carbon soot with fullerene molecules are also obtained.	Various applications, including nanoelectronics reinforcement in composites.	[77]
CNTs	Experimental research	CNTs incorporated into cast steel enhance the productivity of small agricultural machinery castings, leading to increased mechanization levels.	Agriculture, mechanization.	[88]
CNCs	Pulsed laser ablation	1. SnO_2_/CNT nanocomposites effectively degrade methylene blue (MB) dye in photocatalysis. 2. AuNP/CNT nanocomposites demonstrate biocompatibility and toxicity toward cancer cells. 3. Laser processing controls chemical composition and mechanical properties of CNT composites.	Wastewater treatment, cancer therapy, and various applications.	[60]
MWCNTs	Flame fragment deposition (FFD), purification	Handmade reactor and flame fragment deposition (FFD) method synthesis MWCNTs from liquefied petroleum gas. Purification with hydrogen peroxide and acetone yields MWCNTs with varying diameters and purity exceeding 65%.	Various applications including energy storage, nanosensors, and NCs.	[73]
CNTs	Flame synthesis	Flame arrangement significantly influences CNT morphology and crystallinity. Temperature variations in flames produce CNTs with different properties, emphasizing the importance of flame configuration for tailoring CNT characteristics.	Energy storage, nanosensors, NCs.	[74]
CNTs and CNOs	Flame synthesis (alcohol Bunsen burner)	Alcohol flame synthesis using a Bunsen burner produces CNTs and CNOs from n‐butanol and ethanol fuels, influenced by ammonia additives. Flame synthesis offers a facile and efficient alternative to traditional techniques.	Various applications, including nanoelectronics, and reinforcement in composites.	
MXene/CNT‐supported epoxy (EP) composite	Freeze‐drying	MXene/CNT‐supported epoxy (EP) composite exhibits improved flame retardancy and effective EM shielding (34.7 dB). Low MXene/CNT content results in reduced heat release, smoke, CO, and CO_2_ production rates.	Electronics, flame retardancy, electromagnetic interference (EMI) shielding.	[95]
MWCNTs	Hydrothermal Synthesis	Hydrothermal synthesis using iron catalysts and glucose yields homogeneous MWCNTs suitable for various applications.	Various applications.	[93]
CNTs	Hydrothermal Synthesis	Hydrothermal synthesis with sucrose and nickel‐based catalysts allows for control over CNT shape and diameter, promoting sustainability and quality in CNT production.	Various applications.	[94]

### 3.7 Critical Comparison, Scalability, and Quality Control Challenges

Although there are significant advances in the production of CNT, there is not yet a single preparation technique that meets all the demands of narrow structural control, high yield, cost‐efficiency, and scalability to industrial levels. CVD is the most used, with its relatively low cost, its ability to grow continuously and its ability to work with a variety of catalyst systems and reactor designs. Recent studies on CNT synthesis show that CVD can be scaled up, but it often loses control over crucial factors like defect density, chirality specificity, wall number, and diameter distribution, especially in applications with vast areas or high throughput [96, 97]. Conversely, arc discharge and laser ablation techniques can yield CNTs with more crystallinity and a reduced number of intrinsic defects, but with low throughput, high energy usage, limited parameter control, and large‐scale production is difficult [98].

The CNT quality remains a constant problem in all synthesis pathways. Structural flaws, amorphous carbon byproducts, and remaining metal catalysts that are commonly added or amplified in high‐temperature growth or purification may significantly affect electrical conductivity, mechanical strength, thermal stability, and chemical reactivity. These quality concerns are particularly important in the case of laboratory research to a practical device or composite fabrication where reproducibility is crucial [96, 97].

Postsynthesis purification and functionalization methods can be used to increase dispersion and interfacial compatibility, but these methods often add other defects or reduce CNT lengths, compromising intrinsic properties. Even minor changes in the composition of catalysts, growth temperature, reactant concentration, or reaction time can cause very large fluctuations in CNT morphology and activity that restricts their reliable incorporation into high‐performance electronic devices and CNCs [97, 98].

Production of chirality‐specific nanotubes is one of the most important unresolved issues in CNT synthesis. Due to the direct relationship between CNT chirality and electronic behavior, whereby a nanotube is metallic or semiconducting the absence of chirality‐selective growth has been a key roadblock to nanoelectronic, optoelectronic, and sensing devices. Despite these advances in chirality control, such as recent reports of catalysts that allow selective growth of a particular chirality in high purity, such approaches are typically complicated, expensive, or challenging to scale up [96, 99].

As a result, the design of synthesis pathways that can both allow the control of chirality, low levels of defects, and scalable production is a key research focus and a major requirement in the commercial implementation of CNT‐based technologies.

## 4 Properties of CNTs and Their Composites

### 4.1 Electrical Properties

CNTs, and CNCs, are becoming anincreasingly important focus for studying and technological advancement due to the immense electrical properties, i.e., electrical conductivity allows them to carry electricity effectively. Using SWCNT‐based electronic and optoelectronic systems requires a thorough understanding of the interaction between the structural components of SWCNTs and their electrical properties. Examination of eleven different (*n*, *m*) single‐chirality SWCNT films revealed notable differences in carrier mobility and on‐state current, even between SWCNTs with identical diameters but varying chiral angles. Differences in electronic band structures, which affect intrinsic resistance, contact barriers, and intertube contact resistance, are attributed to this variation. Simultaneously, they evaluated three feeding techniques while toughening polypropylene (PP) using a NP‐elastomer blend. The best results were obtained with sequential feeding, which improved MWCNT localization and dispersion, strengthening fracture toughness and increasing impact strength. The sequential approach also made it easier to create electrically conductive channels, enhancing electrical characteristics by bridging NPs and building a network [100, 101]. Density functional theory (DFT) is used to study the stability and tunable bandgaps of 1D pentagonal PdSe_2_ nanotubes (p‐PdSe_2_ NTs) under strain. Structures stay stable under moderate strain, but under high strain, fragmentation happens, which modifies the electrical band structure. High on/off ratios and current modulation points to possible uses in cutting‐edge electronic devices [102].

Furthermore, depending on their chirality, CNTs may be either metallic or semiconducting, and this trait can be used in a variety of electronic applications. Conventional silicon‐based integrated circuits (ICs) have been an essential component of computational electronics for more than 50 years, with a wide range of applications. But as silicon‐based technology approaches its limits, new electronic materials are needed to keep society moving forward. s‐SWCNTs present a possible substitute due to their excellent ballistic transport and carrier mobility. Even with the tremendous advancements in carbon‐based electronics, it is still difficult to fully utilize s‐SWCNTs because of their chiral variety, which causes variations in electrical performance and makes them unsuitable for use in high‐end ICs. This article highlights potential applications while delving into chiral sorting techniques and developments in monochiral CNT FETs [59]. Because of obvious benefits, electrochemically powered actuators which are well‐known for having low operating voltages and controllability are being explored in detail. With the use of hierarchical CNT fiber (CNTF) architecture, Wang et al. created a high‐twist‐pervaded structure for CNTF actuators, which allowed for a significant 62.4% tensile contraction. Thermal actuators, on the other hand, use joule heating to provide quick electrothermal response, making temperature control simple and useful in a variety of applications. In the meantime, interest in 1D fiber‐shaped devices woven into fabrics has increased due to the need for sophisticated electrical energy storage and electronic display devices, notably in portable electronics like smartphones and laptops. Because of their superior mechanical qualities, high electrical conductivity, and lightweight nature, CNTFs are a viable material to use in the fabrication of these types of devices, outperforming traditional fiber electrodes in a range of applications [103].

### 4.2 Thermal Properties

Due to their extraordinary thermal characteristics, CNTs have attracted much interest in both scientific and industrial applications. Their remarkable heat conductivity, which may outperform than those of most materials, including copper and diamond, is one of their most remarkable properties [104]. The number of layers in several types of CNTs varies, including SWCNTs, DWCNTs, and MWCNTs [105].

Melk et al. studied the temperature dependent thermal conductivity of yttria‐doped tetragonal zirconia CNTs (TZ‐3Y‐MWCNTs) composites with various MWCNTs concentrations. The thermal conductivity of obtained samples was negligible at low concentrations of MWCNTs but significantly reduced at higher concentrations. It was found that increasing the temperature of SPS to 1500°C did not show any significant effect on the thermal conductivity of 0.5 and 1wt% CNT compositions. Only a small decrease is observed at high temperature for 1wt% CNT, but one would expect a higher thermal conductivity because of the increase in grain size when SPS is carried out at 1500°C [106]. The thermal conductivity of 3Y‐TZP with a grain size of 179 nm is presented in Table 6. It has also been observed that the thermal conductivity decreases with temperature, with absolute values between 2.83 and 2.03 Wm K^−1^ in the range 100°C–1000°C [107]. Furtherly, Akter et al. focused on epoxy composites with graphene nanoplatelets (GNPs) as fillers, finding that the thermal conductivity was influenced by the loading percentage, filler type, particle characteristics, thermal contact resistance, and dispersion at the polymer–nanofiller interface. They observed that even a small increases in GNP loading (0.1, 0.2, 0.3 wt%) improved the thermal conductivity [108]. In another study, Koltsova et al. found that copper‐based composites with 20–30 nm fullerene exhibited higher thermal conductivity when prepared by molecular level mixing (397 Wm^−1^ K^−1^) compared to mechanical mixing (332 Wm^−1^ K^−1^), due to better structural homogeneity and reduced residual stresses. The small size of the fullerene played a crucial role in achieving thermal conductivities comparable to CNT‐based composites, despite the lower intrinsic conductivity of fullerene, emphasizing the significant impact of particle size and mixing methods on thermal performance [109].

**TABLE 6:** Temperature dependent thermal conductive behavior of CNTs and their composites.

Material composition	MWCNT/other additives concentrations	Temp (*T*), °C	Grain size, nm	Thermal conductivity behavior	Thermal conductivity values	References
Yttria‐doped tetragonal zirconia (TZ‐3Y) with MWCNTs	CNTs addition (0.5, 1 and 4 wt%)	1500	Not specified	Decreases by increasing CNT content and temperature in the range studied	Not specified	[106]
3Y‐TZP and 3Y‐TZP‐CNT	Various (5 wt%)	100–1000	179	Thermal conductivity decreases with temperature	2.83 and 2.03 W m K^−1^ (100–1000°C)	[107]
Epoxy composite with graphene nanoplatelets (GNPs)	Various (0.1, 0.2, 0.3wt% GNPs)	—	—	Increased with GNP addition	0.1283 W/m K	[108]
Copper‐based composites	fullerene soot, up to 23 vol%	—	20–30	Smaller size have greater thermal conductivity	397, 332 Wm^−1^ K^−1^	[109]

Hanzel et al., found a similar effect in Al_2_O_3_‐MWCNTs composites. Single SWCNTs can reach temperatures over ten times higher than copper in terms of longitudinal thermal conductivity [110].

**FIGURE 7:**
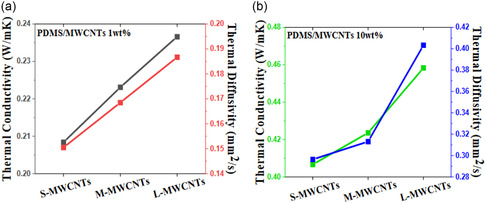
Thermal conductivity and diffusivity data of (a) PDMS/MWCNT 1 wt% composites and (b) 10 wt% composites with three different aspect ratios. Adapted from [112].

Strong covalent carbon–carbon bonding and CNTs 1D structure enable effective heat transport along the tube axis and exceptional overall thermal stability [111]. Additionally, CNTs can improve heat dissipation in electronics and other thermal management applications by acting as good thermal interface materials. This means that CNTs have the potential to address heat‐related issues in modern technology [111]. Lee et al., studied the thermal properties of MWCNT/polymer composites with different aspect ratios to investigate their effects on thermal property by three‐roll milling process. CNTs were uniformly dispersed in the polymer matrix to form a conductive network. They found that the thermal conductivity and diffusivity of the PDMS/MWCNT composites for each aspect ratio showed that L‐MWCNTs composite have the best thermal properties with a high aspect ratio as compared to those with a low aspect ratio shows the trend in Figure 7a,b [112].

CNTs have a high aspect ratio, which makes them useful for reinforcing composites. This improves the mechanical and thermal properties of materials such as ceramics and polymers [113]. The electronic structure of MWCNT is more complex than that of SWCNT because MWCNT is composed of several coaxial SWCNT. These unique thermal qualities have opened a wide range of applications, including efficient heat sinks and novel materials for the electronics and aerospace industries.

### 4.3 Mechanical Properties

Mechanical properties are the physical characteristics that determine how a material reacts to forces from the environment. These characteristics include elastic and Young's modulus, hardness, toughness, tensile, compressive, and flexural strengths. Because of their strong covalent carbon–carbon bonds and distinctive 1D structure, CNTs have exceptional mechanical characteristics. The tensile strength of CNTs can surpass those of most materials, including steel, making them very robust [114]. Defect and impurities reduced the tensile strength in CNT as they disturb the ideal lattice structure of carbon atoms by generating week point alongside CNT that result in further damage under tension. Consequently, as the impurities or defects level increases, the overall tensile strength decreases [115]. A single defect in CNTs can sometimes decrease the tensile strength by 50% which merely depends on type and location of defect as CNT with multiple defects can cause greater reduction in CNTs. The tensile strength is reduced by 26% due to single vacancy defect whereas, topological defect may decrease it by half [116]. Defects and impurities in CNTs significantly decrease their tensile strength, as they disrupt the perfect lattice structure of the carbon atoms, creating weak points along the nanotube which can lead the earlier failure under tension; essentially, the more defects or impurities present, the lower the overall tensile strength of the CNTs.

Because of these characteristics, CNTs serve as the perfect reinforcement for composite materials. In these materials, they improve the stiffness and strength of ceramics, polymers, and other materials. Moreover, CNTs have outstanding flexibility, which allows them to sustain large deformations while maintaining their original shape. Strength, stiffness, and flexibility together have produced a wide range of applications in materials science and nanotechnology, ranging from the creation of innovative sensors and advanced NCs to high‐performance structural materials. The process of making composites heavily depends on dispersion and distribution. When creating interconnected networks, CNTs with uniform distribution and good dispersion are ideal. However, depending on the type of polymer matrix, it might require a certain degree of agglomeration and a well‐planned nonuniform distribution, which could lead to separated structures with better mechanical properties [113]. Continuum modeling is often used to estimate the mechanical properties of CNTs. Rather than considering the individual carbon–carbon bonds, the tube is treated as a continuous structure with uniform mass and material distribution. While validating continuum models takes careful consideration, analytical finite element (FE) methods are often used to examine CNT behavior [117]. Li et al. predicted the mechanical and tensile properties of SWCNTs using a representative volume method based on FE analysis [118]. Kianfar et al. used a 3D FE model to estimate the mechanical and thermal impact properties of coiled CNTs embedded in a matrix, whereas Zhu et al. adjusted the Mori–Tanaka scheme to evaluate the properties of imperfect wavy CNTs [119, 120]. In order to anticipate the mechanical properties of SWCNT‐reinforced composites at the CNT‐matrix interface, Tadi Beni et al. suggested a shell‐based model that uses the principle of minimum potential energy to predict the properties of chiral CNTs [121].

Each study has a different field of focus and has proposed works keeping different views in mind. However, the processing techniques, mechanical testing methods, and mechanical calculations through stress–strain curves are some of the entities which were comparable in the literature available. In all the studies the common target has been the calculation of tensile strength, however, some studies focused on the flexural and short‐beam shear strength of the composites. In a few articles the exact quantity was not quoted, so considering the results, the values were evaluated and summarized.

**FIGURE 8:**
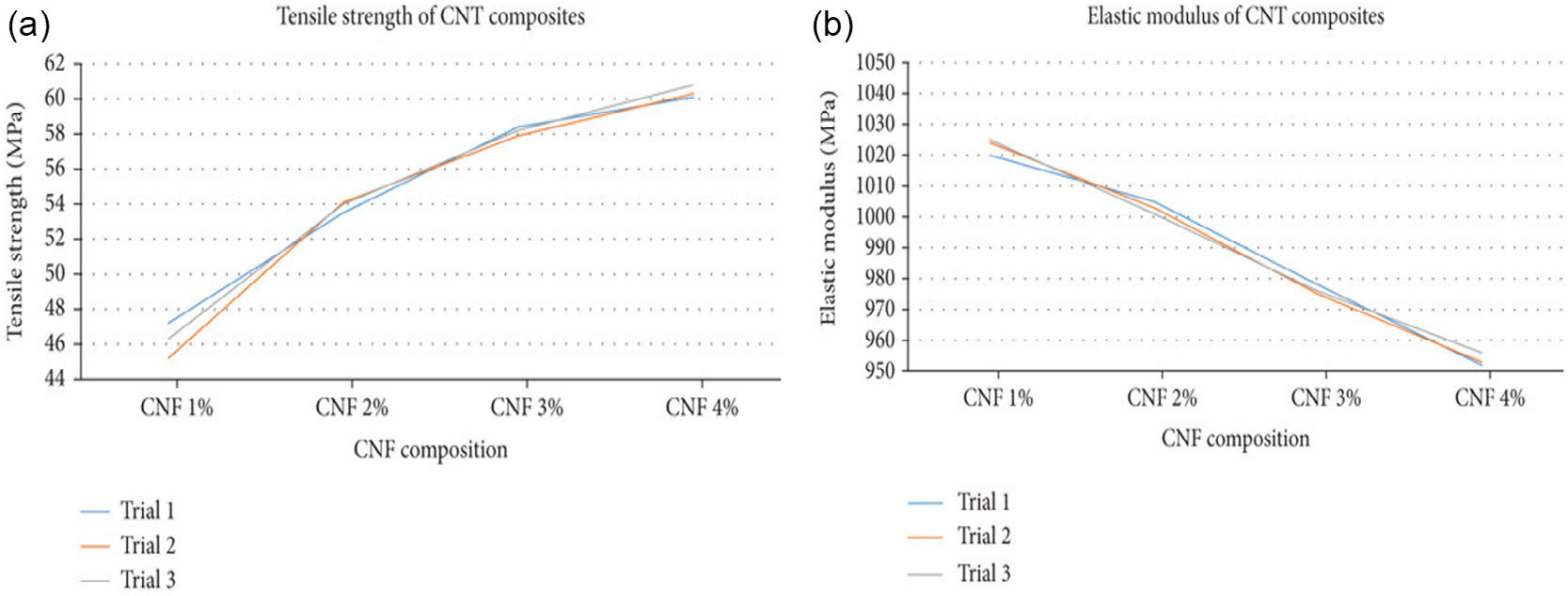
(a) Shows the graphical presentation of the tensile strength of the CNT composites developed. (b) Hardness for CNT composites. Adapted from [101].

CNTs were utilized by Merneedi et al. to build the composite with the resin. The best proportion for the best mechanical properties is selected when using CNT as a filler material in the composite; the range of percentages is 1%–4%. Although the rate of increase slows down with time, the tensile strength rises as the concentration of CNTs does. Tensile strength measurements for CNT with a 4 wt% composite were 27% higher than those for CNF composite, which was 1% shown in Figure 8a,b [122].

**FIGURE 9:**
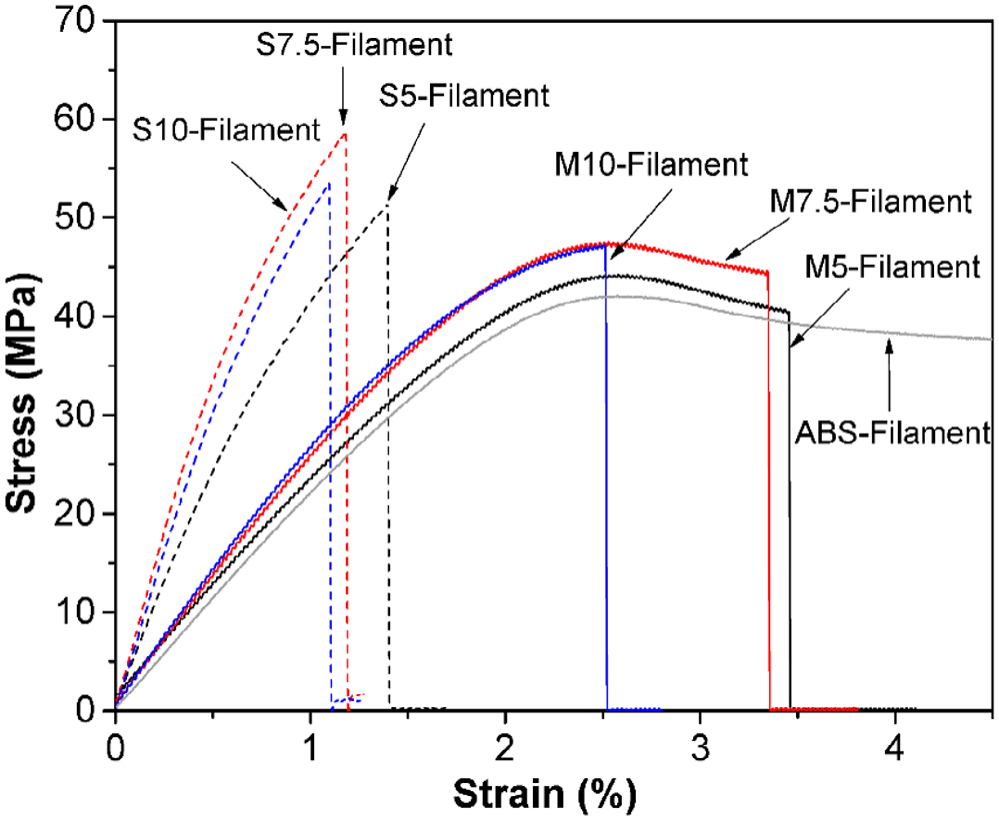
Representative stress–strain curves of ABS filament and nanocomposites filaments containing MWCNT (line) and SWCNT (dot line) up to 10 wt%. Adapted from [102].

The acrylonitrile‐butadiene‐styrene (ABS) filled‐CNT nanocomposites that Gutierrez et al. investigated were correctly created by a solvent‐free procedure in blend compounding at 190°C. These CNCs had different filler loadings of 5–10 wt%. Extruded filaments and compression‐molded plates were produced at 190°C and 230°C, respectively. The effect of MWCNT and SWCNT reinforcement on the mechanical characteristics of filament samples is examined in ABS nanocomposites. The elastic modulus of SWCNT‐based nanocomposites is higher than that of MWCNT‐based composites, according to the stress–strain curves shown in Figure 9. For instance, the nanocomposite containing 10 wt% SWCNT showed an increase in elastic modulus from 2207 to 6190 MPa (or about 280%), while the similar result for the nanocomposite containing 10 wt% MWCNT is only 2771 MPa (or roughly 26%). The stiffness, shape, orientation, and dispersion level of the nanofiller have an impact on the nanocomposites’ elastic modulus [123].

In a follow‐up work from 2022, Mateab and his coworker utilized the solution mixing procedure to achieve improved dispersion with a probe tip. The varying epoxy nanocomposites’ hardness (shore‐D). A little increase in hardness by increasingthe microparticle concentration, i.e. epoxy‐MWCNTs ratio (0.25–2 wt%) showed an improved impact strength of around (33.3%, 45.8%, 75%, and 107.5%) of obtained MWCNTs/epoxy composites. The percentage of hardness increased from 0.41 to 3.12 as well [124]. These results highlight how crucial it is to optimize the type and concentration of CNTs for certain mechanical properties in composite materials.

### 4.4 Magnetic Properties

**FIGURE 10:**
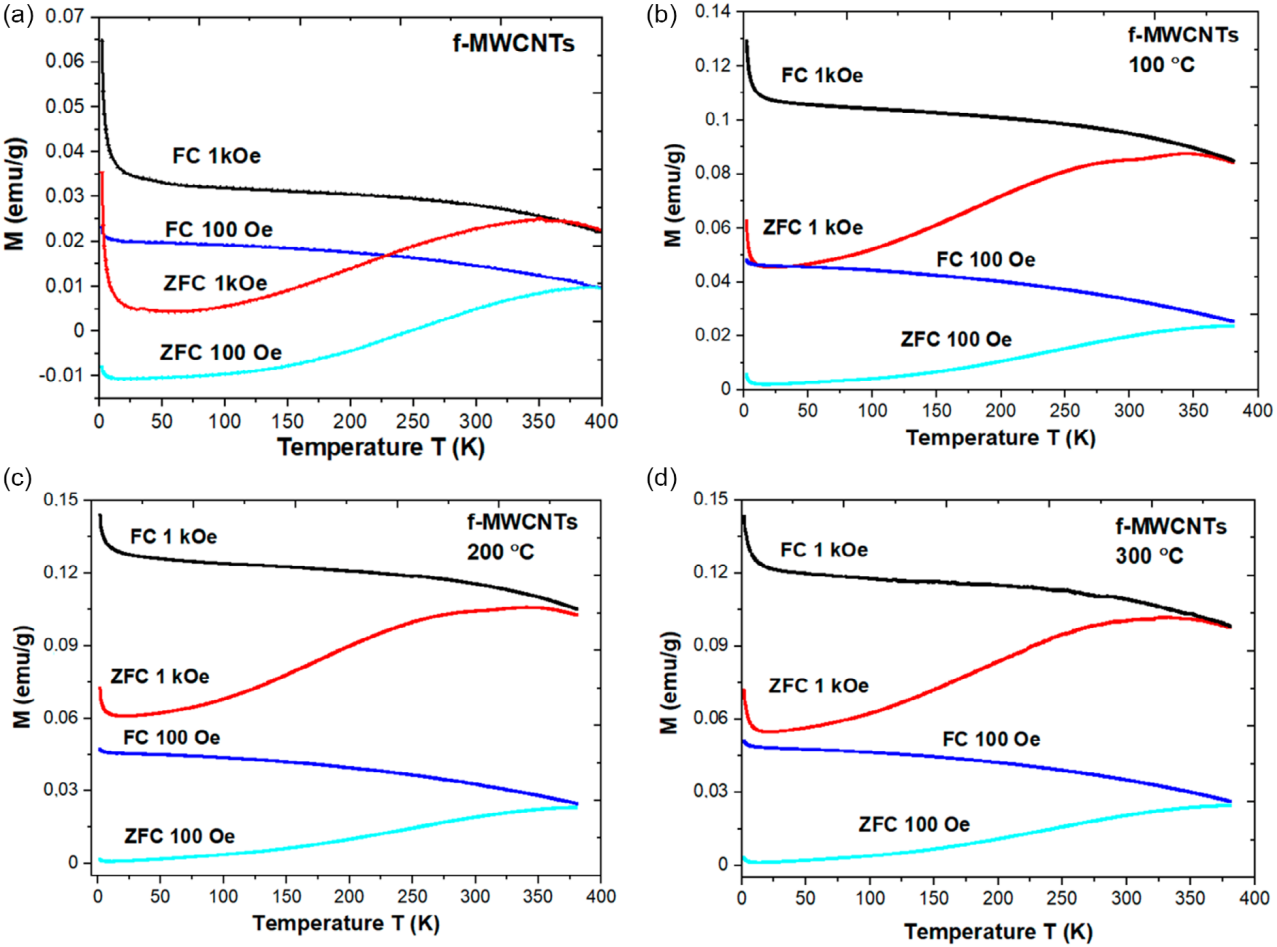
Temperature dependence of magnetization for calcined f‐MWCNTs (a) pristine, (b) 100°C, (c) 200°C, and (d) 300°C [135].

**FIGURE 11:**
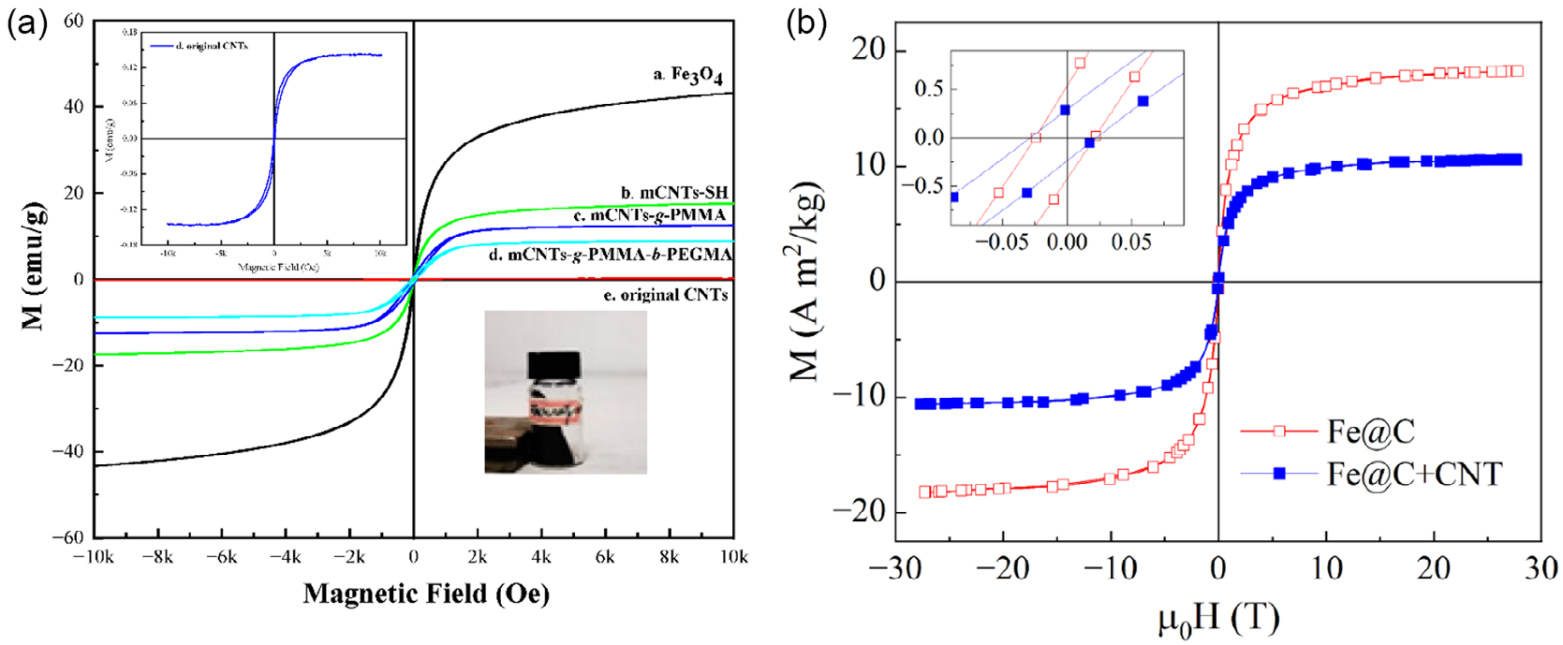
Magnetic hysteresis loop of carbon nanotubes and composite (a) composite membrane with CTNs [137]. (b) Composites containing Fe@C particles and Fe@C + CNT [138].

The magnetic characteristics of CNTs are fascinating and have garnered much interest in both basic study and practical applications. The primary causes of CNTs’ magnetic behavior are imperfections on their surface or unpaired electrons at the tube ends. The CNTs are magnetic by nature because of their unpaired electrons, which produce localized magnetic moments. Magnetism in CNT can also be due to metal impurities left in it during preparation [125]. CNTs prepared by the CVD contain Fe, which contributes to magnetic properties in CNTs [126]. Besides this, when CNTs are placed in an external magnetic field, they exhibit magnetic properties. Magnetic functionalization of CNTs can also be achieved by its integration with magnetic materials, which leads to the formation of magnetic CNCs [127]. CNTs’ magnetic properties have been investigated as potential for developing magnetically functional nanocomposites, spintronics [128] and nanoscale sensors. Mekuria et al. studied the synthesis of a nanocomposite made of cobalt ferrite (CFO) magnetic NPs and CNTs using a one‐step method that included mixing and sonication in the presence of a surfactant. Vibrating sample magnetometer (VSM) and magnetic force microscopy (MFM) techniques were used to assess magnetic characteristics. As a significant improvement, CNTs had strong saturation magnetization (Ms) of 207.7 emu/g (compared to 63 emu/g in pure CFO [129], indicating that magnetic parameters of CNTs could be manipulated at express terms of doping, defect control, and synthesis techniques. The bifunctional PPTA fibers were excellent compared to PSQ‐modified fibers (having single functional groups of either ‐NH_2_ or ‐SH) in the adsorption of Hg(II) [130]. In the same manner, the preparation of magnetite‐silica adsorbents containing the amino and mercapto groups provided excellent responses to Ag(I), Cu(II), and Pb(II) by Melnyk et al. [131]. The works of Bajorek et al., Jamrozik et al., and Song et al. highlighted the idea of the sensitivity of the magnetic behavior of CNTs to the modes of synthesis and modification [132, 133, 134]. The improvement in magnetization was observed by Bajorek et al. following the calcination process, which was attributed to factors including thermal blocking effects due to magnetic anisotropy and the presence of other Fe‐based species. These findings have high significance for the design of superior materials and applications, as they provide a deeper understanding of the factors that affect the magnetic behavior of CNTs. Bajorek et al. examined the thermal process of magnetization evolution of calcined and pristine nanotubes (Figure 10) [135]. They observed that the calcination process had a minor influence on the difference between field‐cooled (FC) and zero‐FC (ZFC) magnetization, which, however, elevated the magnetization. They also found thermal blocking effects that might be linked to the presence of more than one Fe‐based species due to magnetic anisotropy. On the other hand, Jamrozik and colleagues examined how grinding process affects the magnetic properties of MWCNTs. They found that grinding, especially in a steel mill, increase the superparamagnetic phase's contribution, notably in functionalized MWCNTs. They also noticed nonzero coercivity at room temperature, suggesting sizable ferromagnetic contributions. In their investigation into the functionalization of magnetic CNTs [136]. Song et al. observed that all samples showed superparamagnetic behavior with 0% residual coercivity and field strength. Due to a decrease in the mass ratio of magnetic particles, the saturation magnetic induction strength of magnetic CNTs‐SH decreased with polymer grafting, as shown in Figure 11a [137]. In contrast, Rinkevich et al. focused on a NCs made from a mixture of Fe@C particles and CNTs. They prepared the composite by blending Fe@C powder with CNTs in epoxy resin, followed by solidification. Their magnetic measurements, conducted up to 28 kOe at room temperature, showed the magnetic behavior of the Fe@C particles and the Fe@C + CNT nanocomposite (Figure 11b) [138]. Both studies highlight the enhanced electromagnetic properties of magnetic materials achieved by combining with CNTs, even different methods and materials were used in their studies.

**FIGURE 12:**
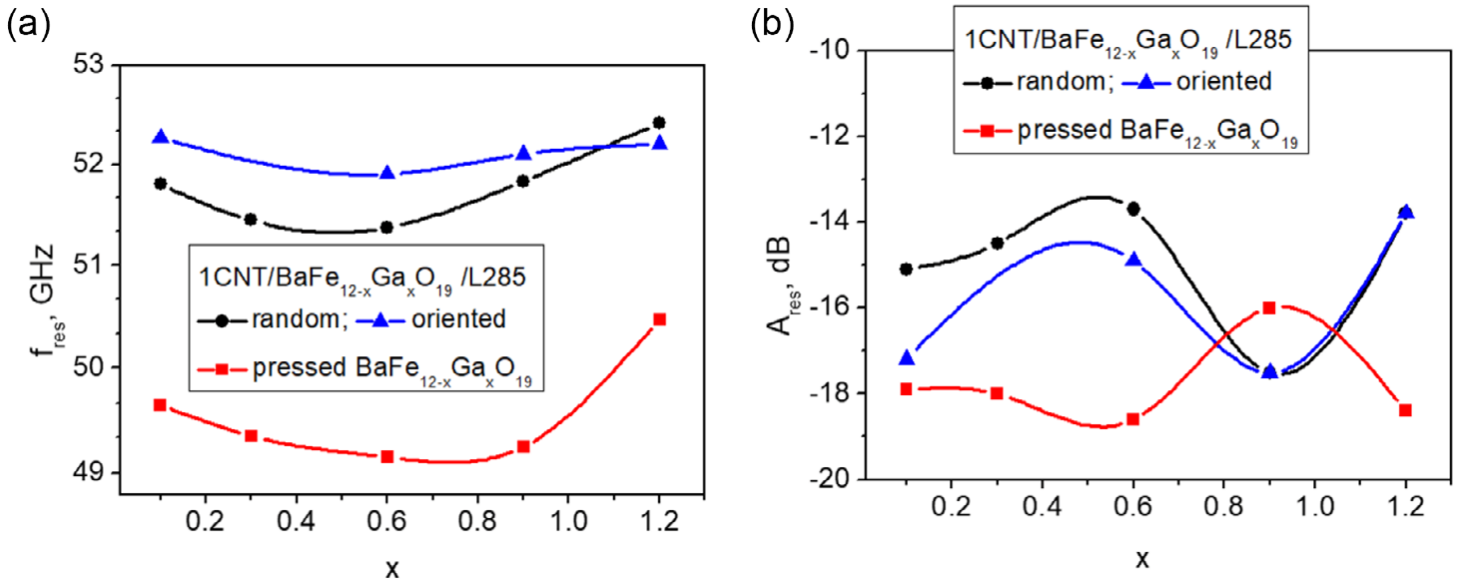
Resonant frequency of NFMR with varying concentration of BaFe_12–*x*_Ga_*x*_O_19_/epoxy composite [139].

Yakovenko and his team investigated the microwave shielding efficiency of epoxy composites filled with 30 wt% BaFe_12–*x*_Ga_*x*_O_19_ and 1 wt% CNTs in the 36–55 GHz frequency range. They found that adding CNTs to the BaFe_12–*x*_Ga_*x*_O_19_/epoxy composite significantly enhance the microwave shielding due to dielectric mixing, magnetic losses, and the extended range of effective electromagnetic radiation attenuation. The resonance frequency of NFMR was also higher in the ternary composites compared to the BaFe_12–*x*_Ga_*x*_O_19_ alone (Figure 12a,b) [139].

### 4.5 Optical Properties

CNTs are of great scientific interest due to their amazing optical traits, which have opened a wide range of applications in several sectors. The broad spectrum of light that CNTs can absorb and emit makes it one of its most unique optical characteristics. Strong light absorption is a well‐known property of CNTs, especially in the visible, near‐infrared, and ultraviolet (UV) spectrums [140].

For owing fantastic optical properties, CNTs have applications in sensors, photodetectors, and light‐absorbing components in various optoelectronic systems. Additionally, CNTs have significant photoluminescence, which allows for the customization of their optical characteristics since various chiralities produce light at different wavelengths [141]. Chiral carbon dots are luminescent carbon NPs that have attracted much attention in the biomedical domain because of their low toxicity and ease of synthesis, especially when they possess chiral characteristics. Novel chiral carbon dots with attractive optical properties, such as two‐photon absorption and circularly polarized light emission, have been created through advances in synthetic chemistry. Chiral carbon dots exhibit absorbance and photoluminescence (PL) properties that are quite similar to those of their achiral counterparts manufactured using comparable techniques. Absorption spectra of chiral carbon dots often show bands at 240–270 nm, which are representative of the *π*–π* transitions occurring within the sp^2^‐hybridized carbon core [142, 143]. Furthermore, it is typical to see absorption peaks in the 300–350 nm region, which are attributed to carboxyl groups or n–π* transitions [144].

Remarkably, Ru et al. found absorption bands for chiral carbon dots that emit red light at 535 nm and yellow light at 425 nm, respectively. These bands were connected to transitions related to surface states [145]. CNTs are considered to be attractive prospects for new photonics and optoelectronics technologies due to their unique optical characteristics and their flexibility to be functionalized or incorporated into diverse composite materials [146]. Novel applications in fields including imaging, light harvesting devices, and telecommunications are continually being inspired by the investigation of the optical characteristics of CNTs.

### 4.6 Surface Properties/Wettability Characteristics

**FIGURE 13:**
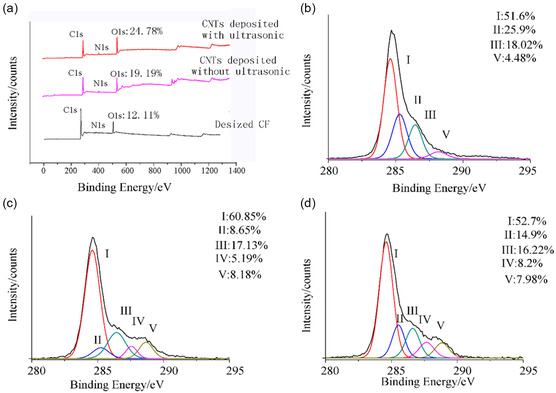
XPS analysis of CNTs‐CF (a) wide‐scan spectra, (b) C1s spectra of desized CF, (c) without ultrasonic; and (d) with ultrasonic [155].

**FIGURE 14:**
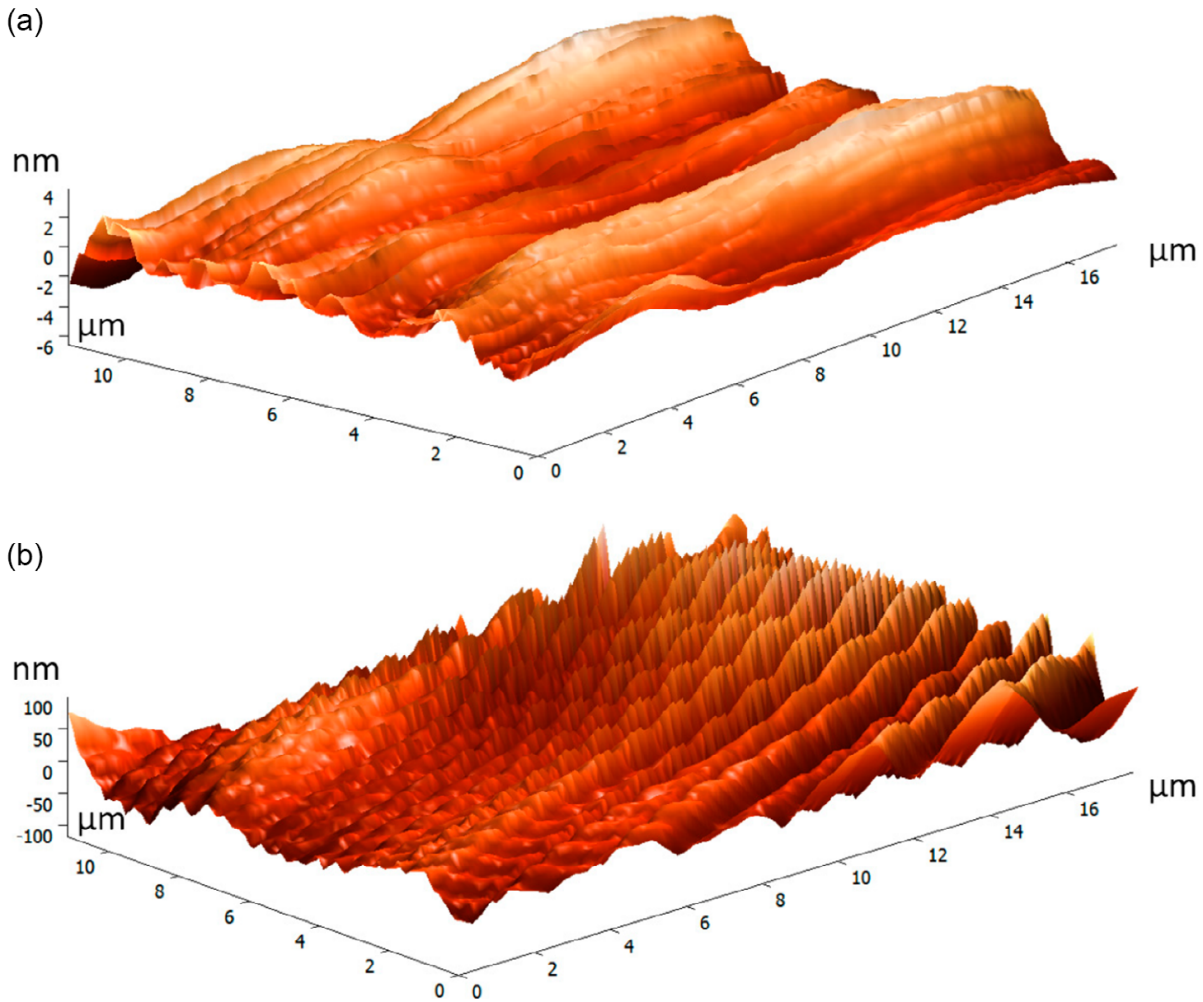
3‐D AFM images of pure (a) Cu and (b) Cu structured with CNTs [156].

CNTs are useful in a variety of industries due to their unique wettability and surface characteristics. CNTs surfaces are hydrophobic by nature because they lack polar functional groups and have strong sp^2^ carbon–carbon bonds. Applications that need low surface energy or water repellence may benefit from this hydrophobicity. In other instances, though, such as environmental, and biological applications, it might also provide difficulties since CNTs might need to be made more hydrophilic to improve compatibility. The wettability of CNTs has been changed by a variety of surface modification methods, such as functionalization with polar groups (such as ‐COOH, ‐OH), plasma treatment, and coating with surfactants [147, 148]. Because of their strong mechanical characteristics and large porosity, meshes have been the subject of most previous studies on superhydrophobic surfaces [149, 150, 151]. Li et al.'s work showed that superhydrophobic absorbents could be made solely from polymers, but the complex chemical reaction and fluorine content presented serious problems [152]. Meshes supported by materials like stainless steel and copper supported by polymers have been the subject of several research, which have shown good stability and reusability in corrosive settings [18, 153]. Moreover, CNTs’ surface chemistry can be harnessed for applications such as gas adsorption and separation, where tailored surface functionalization can enhance their selectivity and adsorption capacity [154]. The ability to manipulate the surface properties and wettability of CNTs underscores their versatility and wide‐ranging utility in a variety of technological and scientific domains. Jiang et al. and Kamanina et al. investigated the effects of CNTs on different surfaces, but with distinct focuses. Jiang et al. observed that after depositing CNTs on carbon fibers (CNTs‐CF) using ultrasonic techniques, there was a sharp decline in carbon concentration and a significant increase in oxygenated groups on the CF surfaces, suggesting the introduction of polar groups by the CNTs. The use of ultrasonication during the electrophoretic deposition process also increased the amount of CNT coatings on the CF surfaces (Figure 13) [155]. Kamanina et al. studied the topography of a copper surface after CNT deposition, using 3D‐AFM to reveal an irregular surface with vertically oriented CNTs. They noted an almost‐homogeneous distribution of CNTs, with the thin film thickness ranging from 50 to 100 nm, though some areas reached up to 150 nm due to variations in CNT length. The detailed visualization of CNTs on the matrix surface was highlighted as crucial for advancing novel composite structures (Figure 14) [156]. Both studies emphasize the impact of CNT deposition on surface properties, enhancing functionalization and structure development.

### 4.7 Modeling Based on Other Properties of CNTs and Their Composites

Many researchers have reported on modeling based on additional properties of CNTs and their composites, all of which emphasize the materials’ versatility and potential applications. Niidome et al. investigated the structural separation, biosensor applications of SWCNTs, and solubility using polymer wrapping techniques. By modifying the local polarity and hydrophobicity at defect sites, polymer side chains and substituent structures affect the E11* PL shifts of locally functionalized SWCNTs (lf‐SWCNTs) in response to changes in the dielectric environment. This article demonstrates that the interaction of molecules can be highly controlled with the aid of SWCNTs to create advanced sensors [157]. In contrast, Dr. Mark Wilson examined how Monte Carlo simulations can be used to investigate the mechanical susceptibility of MWCNTs in ferromagnetic composites. The results showed an improvement in magnetic characteristics that can be utilized in spintronic devices, resulting in enhanced magnetic storage density [158]. Saya et al. used the characteristics of several chemical sensors to identify the nerve agent simulant dimethyl methyl‐phosphonate (DMMP). Their research highlighted the significance of nanomaterials in the advancement of sensors, including chemi resistors, FETs, chemi capacitors, and mass‐sensitive sensors. The work involved a thorough analysis of how the characteristics of nanomaterials enhance the sensitivity and reaction time of gas sensors for detecting DMMP [159]. In the meantime, Bamane et al. investigated the wettability of several epoxy systems on boron nitride nanotube (BNNT) surfaces using molecular dynamics (MD) simulations. The results showed that temperature and epoxy type had a substantial impact on resin infusion for high‐performance BNNT/epoxy NCs [160]. Raj et al. investigated the mechanical properties of PMMA composites enhanced with SWCNTs using MD simulations. They discovered that the mechanical properties are greatly improved by varying the volume fractions, temperatures, and CNT sizes, with DWCNTs exhibiting the best tensile strength [161]. The two studies demonstrate how nanotube‐reinforced composites can be optimized for innovative applications using MD simulations.

### 4.8 Advanced Characterization Techniques for CNTs and CNT Composites

Even though scanning electron microsscopy (SEM) and transmission electron microscopy (TEM) have been described as standard methods of morphological characterization in CNT research, recent work indicates that sophisticated methods of characterization are necessary to develop sound structure‐property‐performance relationships in CNTs and CNCs. In‐depth analysis of the literature indicates that surface analysis, mechanical analysis, high‐resolution imaging, and surface chemistry characterization methods are essential in confirming the functionality of CNTs in energy, structural, and sensing applications [162, 163]. BET surface area analysis is especially useful when it comes to assessing CNTs as energy storage media as well as catalysts. It has been shown in recent reports that increasing accessible surface area and maximizing pore size distribution is highly important in increasing ion transport and charge storage in CNT‐based supercapacitors and hybrid electrodes [164, 165]. Yahyazadeh et al. also observed that poor dispersion and aggregation may severely decrease effective surface area, despite CNTs having theoretically high values [163]. Although this is relevant, the reporting of BET analysis remains inconsistent and as such it is not possible to directly compare electrochemical performance of various CNT synthesis and composite fabrication strategies.

Dynamic mechanical analysis (DMA) is becoming a highly important instrument in establishing the reinforcing efficiency of CNTs in polymer and hybrid composites. The recent experimental research (Wang et al*.*, 2024) has shown that DMA provides the possibility to directly measure the efficiency of load transfer, interfacial bonding, and viscoelastic performance of CNT‐reinforced epoxy systems [166]. The results of the studies prove that the quality of dispersion of CNTs and interfacial interactions are significant determinants of the improvement of storage modulus and damping behavior. In the absence of DMA, it is difficult to differentiate between true nanoscale reinforcement and what seems to be mechanical reinforcement due to effects of processing or filler agglomeration.

High‐resolution imaging methods, such as improved SEM and high‐resolution TEM (HR‐TEM), are still essential for determining CNT wall number, diameter distribution, alignment, and defect density. According to recent evaluations, HR‐TEM offers clear evidence of defect generation, catalyst residue encapsulation, and lattice integrityall of which have a significant impact on electrical and thermal transport parameters [162]. When comparing CNTs produced using various methods (such as arc discharge vs. CVD) or assessing structural degradation after purification and functionalization procedures, these evaluations are particularly crucial.

The most important technique to confirm the effectiveness of CNT surface chemistry and functionalization is the X‐ray photoelectron spectroscopy (XPS). Recent investigations on CNT surface modification (Aasa et al., 2026 [167]) have shown that XPS makes it possible to quantitatively detect oxygen‐ and nitrogen‐containing functional groups, which gives direct evidence of the oxidation, amination, or heteroatom doping process [167]. This fact is essential in applications of interfacial interactions, including polymer reinforcement, catalysis, and biosensing. Nonetheless, qualitative reporting of XPS data remains common in many studies, and it is not correlated with any functional performance metrics.

In general, the recent literature suggests that the lack of and limited application of sophisticated characterization methods is a significant bottleneck in CNT studies. When incorporated systematically into the research framework of CNT and CNC, BET, DMA, HR‐TEM/SEM, and XPS would play a vital role in enhancing the reproducibility and cross‐study comparability of the results, as well as improve the speed at which CNT‐based materials can make the transition between laboratory‐scale research and real‐world use.

## 5 Applications of CNTs and Their Composites

CNTs are extremely attractive for various applications due to their exceptional mechanical, electrical, and thermal characteristics. While there are many applications for nanotechnology and a constant stream of new materials being developed, CNTs are the most promising. Since 1991, the development of CNT‐based nanomaterials has accelerated due to their exceptional mechanical, electrical, and electron transport capabilities. Although, CNTs have many potential uses, their poor solubility in organic and aqueous solvents has restricted their use. The present work focuses on investigating CNT‐reinforced polymer composites using covalent and noncovalent functionalization techniques. These composites include SWCNTs and MWCNTs [168]. This review evaluates the development of CNTs/ CNCs critically and identifies the most noteworthy advancements in the field. A detailed discussion of a few applications of CNT and their composites have been observed in practical sectors of electronics and sensors, energy storage devices, membranes, engineering structures, aerospace/automobile, and biomedical fields.

### 5.1 Electronics and Nanotechnology

The incorporation of CNTs into a wide range of electrical products has revolutionized industry. The applications of CNTs in field emission, data storage, energy storage, biosensing, nanoelectromechanical systems, and supercapacitors are reviewed in this section. These developments demonstrate the CNTs potential to improve performance and enable the miniaturization of electronic components.

### 5.2 In Electronic Devices as Field‐Emission Sources

CNTs and CNTCs are frequently used in electronic devices for field emission. Their unique structural and electrical properties make CNTs excellent field emitters because they can emit electrons when subjected to a high electric field. Electronic instruments such as electron microscopes, X‐ray sources, and field‐emission screens exploit this application. Since CNTs can emit electrons efficiently, they are useful parts of high‐performance electronic devices that help improve technology and reduce the size of equipment [169], TEM [170], and AFM. Highlighted as superior electron emitters are CNTs, which have low emission threshold potentials, large current densities, extended component lifetimes, and steady field emission over time [171]. Comparing CNTs to traditional components such as anisotropically etched silicon and tungsten, they show a threshold voltage for field emission lower and a current density as high as 4 A/cm^2^ [172]. Equation (1) also states that the chiral angle determines the voltage direction and that the diameter of CNTs can regulate their threshold voltage [173].

(1) $$V_{\mathrm{th}}=\frac{\alpha V_{\pi }}{\sqrt{3}qD_{\mathrm{CNT}}}$$

Numerous scholars have produced noteworthy advancements in the study of CNTs and their composites, with varying emphasis on the synthesis, characteristics, and uses of CNTs. Mehdi et al. assessed the field emission characteristics of MWCNTs synthesized using various techniques, whereas Huang et al. investigated the impact of metal catalyst selection on process yield. Wang et al. created SWCNT‐reinforced copper matrix composites, Kumaresea et al. researched the manufacturing of manganese‐based NPs using thermal plasma arc discharge, and Mohajer et al. investigated the many applications of MXene‐CNT composites [174, 175, 176, 177, 178]. Furthermore, Chang et al. created MXene/CNT‐supported epoxy composites for flame retardancy and EMI shielding [95], Lin Hsu et al. studied the in situ synthesis of CNTs using alcohol flames [179] and Madhurima et al. refined arc discharge synthesis parameters [180]. All these studies advance our knowledge of CNTs and their composites and how to use them in different industries. Because of their exceptional mechanical strength and electron transport characteristics, CNTs are particularly well‐suited for electronic devices. As such, they can be used for electron sources in vacuum microelectronics and field‐emission displays [174, 176].

### 5.3 Energy Storage and Conversion

CNTs have a major influence on the energy industry, especially in batteries, supercapacitors, and fuel cells, as the header illustrates. The article explains how CNTs enhance the effectiveness and efficiency of energy conversion and storage technologies, hence facilitating the creation of sustainable energy solutions. Thus, it highlights CNTs as a flexible material with a wide variety of uses, and it cites CNTs, especially the creation of flexible secondary alkaline batteries that have rechargeability equivalent to that of conventional secondary alkaline batteries [181].

Furthermore, CNT‐based composites exhibit strong potential in energy‐related applications like solar cells, where they improve mechanical stability and conductivity, resulting in increased device longevity and efficiency. Additionally, research on the use of CNT‐reinforced composites in wearable technology, flexible electronics, and sensors has demonstrated the versatility and adaptability of these materials across various industries. In general, incorporating CNTs into composite materials holds great promise for expanding multiple technical domains and addressing challenging engineering issues in the pursuit of sustainable solutions [182]. The latest advancements focus on studying the multifaceted uses of CNTs, as well as their composites, in energy storage and conversion technologies. For example, Chen et al. demonstrated the hydrothermal synthesis of MWCNTs using iron catalysts and glucose, providing a more environmentally friendly method for producing CNTs [183]. By covering paraffin wax (PW) with MoS_2_@CNTs core‐sheath structures, Liu et al. synthesized a multifunctional nanocomposite that integrates solar thermal energy harvesting and microwave absorption. The in situ growth of 2D MoS_2_ on 1D CNTs enhanced photon trapping and phonon transport, resulting in a solar‐thermal conversion efficiency of 94.97% and a phase change enthalpy of 101.60 J/g. The composite demonstrated exceptional thermal durability as well as strong microwave absorption (−28 dB at 12.91 GHz), providing advanced thermal management and energy storage solutions [184]. Additionally, Huang et al. described how to prepare few‐layer FePSe_3_‐CNT (f‐FePSe_3_/CNT) hybrids using a mechanical exfoliation technique as efficient K^+^ storage anodes for potassium‐ion batteries. Tightly coupled 1D‐2D hybrids facilitate potassium ion reaction and diffusion, exhibiting high capacity (472.1 mA h g^−1^), rate (124.9 mA h g^−1^), and cycling stability (>1000 cycles). Full cells of f‐FePSe^3^/CNT anodes connected with a potassium‐ion battery show good energy/power density (54.7 W h kg^−1^/5790.8 W kg^−1^) and strong cycling stability (500 cycles), indicating potential uses in K^+^‐based storage systems. This work provides a general approach to improving K^+^ electrochemical performance in different layered materials [185].

### 5.4 CNTs in Supercapacitors and Actuators

CNTs and CNTCs are used in supercapacitors and actuators because of their remarkable electrical, mechanical, and structural properties. These materials provide high energy and power densities, rapid charge/discharge rates, and prolonged cycling stability. CNTCsalso demonstrate excellent electromechanical capabilities, allowing precise control and efficient energy conversion. CNT‐based electrodes satisfy the need for efficient energy storage devices. Energy storage and actuation technologies could be greatly advanced by using CNTs and their composites in super‐capacitors and actuators [186, 187].

Considering a battery and a capacitor is the simplest approach to describe the two techniques for storing electrical energy. A Faradaic reaction occurs in a battery when two electroactive species are reduced and oxidized, generating chemical energy that powers the device. Electrostatic forces separate the charge in a capacitor by separating oppositely charged plates. Potential is created between the plates by the excess and lack of electron charges without any charge transfer [188].

The main goal of this innovative study was to take advantage of CNTs unique properties for supercapacitor applications. Through the use of CNT electrodes, researchers were able to demonstrate the efficient charge storage capacity of CNTs and obtain a significant improvement in energy storage capabilities [188]. Furthermore, composite electrode materials made of MWNTs covered with polypyrrole were reported to demonstrate a high capacitance of 587 F/g in an acetonitrile/0.1 M NaClO_4_ electrolyte in a recent work by Hu et al. [190].

The 2023 study by Waris et al. investigates asymmetric supercapacitors, which combine the benefits of batteries and supercapacitors to provide increased energy density, enhanced cycle stability, and specific power. The process involved the production of nickel cobalt sulfide (NiCoS) and physically combining it with CNTs. In an asymmetric device, the resulting NiCoS/CNTs composite achieves 161.3 C g^−1^ specific capacity, with a specific capacity of 1542.1 C g^−1^ at 2.5 A g^−1^ in a three‐electrode arrangement. With 84.0% capacity retention after 5000 cycles, it exhibits an impressive 35.5 Wh kg^−1^ energy density and 1800 W kg^−1^ power density, suggesting its potential for high‐performance energy storage systems [189]. Moreover, Zhang et al. used a NiCo Prussian blue analog (PBA) precursor to create armour‐like nanomaterials, namely Ni/Co enclosed by N‐doped CNTs (NCNTs‐Ni/Co). Using the Schiff base condensation process, Mohamed et al. synthesized a porous organic polymer (CE‐Py POP) that included pyrene and crown ether. The formation of a CE‐Py POP/SWCNT nanocomposite involved blending CE‐Py POP with SWCNTs. The CE‐Py POP/SWCNT nanocomposite demonstrated remarkable capacitance retention, retaining 97.6% of its initial capacitance after over 2000 cycles, and a high specific capacitance of 346 F g^−1^ in galvanostatic charge–discharge experiments, suggesting its potential for supercapacitor applications [190].

### 5.5 Aerospace and Materials Science

The lightweight and high‐strength properties of CNTs have revolutionized the creation of innovative materials in aerospace applications. This section examines the use of CNCs to make lighter and stronger aerospace and aviation components, with an emphasis on new developments in structural materials.

No doubt, steel automobile parts offer excellent strength and corrosion resistance. However, high‐performance cars require a reasonably low weight‐to‐power ratio. One can that ceramics could be a viable alternative due to their superior thermo‐mechanical properties and reduced weight‐to‐thrust ratio. Nonetheless, ceramics show their shortcomings for being more brittle and have a shorter lifespan compared to steel alloys [191]. However, ceramics need a possible reinforcement to be used in the aerospace sectors because of their lower fracture toughness and inability to meet the necessary strength. By adding CNT reinforcements to the ceramics, the mechanical and thermal characteristics of the composites could be improved. Additionally, the composites may have a high strength‐to‐weight ratio and fracture toughness, both of which are essential for aerospace applications. In addition to its structural strength, CNTs may protect spacecraft against radiation [192].

Due to the remarkable electrical, optical, and thermal properties of CNTs, researchers are exploring CNTs as potential substitutes for conventional silicon technology. The work focuses on investigating the electrical characteristics of CNTFETs using numerical simulation models. The study focuses on the input–output properties of CNTFETs in ballistic conditions, specifically investigating the effects of diameter, thickness, and temperature [193]. Additionally, Ariharan [194] has conducted research on the suitability of ceramic/CNT composites as bond layer materials and thermal barrier coatings. Goyal et al. conducted research on Cr_2_O_3_ composite coatings with CNTs for use as a thermal power plant barrier. They demonstrated that increasing the CNT content from 1 to 8 wt% resulted in improved corrosion resistance and reduced weight gain. Goyal and his colleagues also reported similar findings regarding the working temperatures of the thermal barriers, which were up to 900°C. With the increase in CNT concentration, corrosion resistance improved while weight gain diminished [195].

### 5.6 Biomedical and Biosensing Applications

CNT and composite production have greatly revolutionized biosensing and biomedical applications, allowing for new approaches in disease diagnosis, tissue engineering, and drug delivery. Current studies by Mathew et al. illustrate the wide promise of MXenes and their composites, especially MXene‐CNT hybrids, which have augmented mechanical, electrical, and environmental stability positioning them as viable options for biosensing devices and biomedical implants [196]. Moreover, in their work, Keihan et al. have revealed the significant potential of the material based on CNTs in biomedical fields, including drug delivery and bioimaging, by employing a simple method of in situ chemical synthesis of CNTs on alloy powder. These conclusions demonstrate that CNTs and their composites have increasing significance in biosensing and biomedical applications, which may offer new routes to disease diagnosis and treatment in the future [197]. Anzar et al. noted that CNTs are versatile due to their large surface area‐to‐volume ratio. These are applied in tissue engineering due to their distinctive mechanical characteristics: they stimulate cell division and regeneration, have a supersaturated drug loading, and can effectively release drugs in an organism. Due to their small size, CNTs can be utilized in biosensors and bioimaging systems to track and detect cells or biomolecules continuously. For example, MWCNTs possess distinct features that are attributed to their sidewalls and end caps, whereas SWCNTs are shaped like hexagonal tubes [198]. Comparatively, the outer diameter of the MWCNTs approximates to 2–30 nm and the inner diameter to 0.3–20 nm. They are composed of 2–50 graphene sheets that have been rolled cylindrically in various configurations. This results in a slightly complicated structure. These measurements work well as scaffolds to assist in the formation and growth of new tissues [199, 200]. Additionally, they may be utilized to directly provide medications and other therapeutic agents to the intended region, enabling a controlled and localized release. Furthermore, it has been demonstrated that CNTs possess antimicrobial properties, which may help avoid infection during tissue regeneration procedures [201]. Safaee et al. developed a unique wearable optical microfibrous textile that they used as a sensing layer to detect oxidative stress in wounds. They did this by encapsulating ssDNA‐SWCNT nanosensors into individual microfibers using a one‐step coaxial electrospinning method [202]. Researchers worked on developing nanotube PNCs utilizing an increasing number of biocompatible polymers and evaluating their function in a range of biological applications in recent articles published in 2023. Xing and his colleagues investigate the PNC as a COVID‐19 viral biosensing instrument [203]. The produced CMP‐CNTs have notable efficiency in photoelectric conversion. The results demonstrated that the suggested photoelectrochemical biosensor has a detection limit of 33 Atto‐mole (aM) and a high sensitivity to the COVID‐19N‐Gene. Through biological analysis and early clinical diagnosis, this created a new avenue for the development of a reliable, sensitive, and easy‐to‐use sensing platform with great potential application. Shen et al. created a flexible biosensor composed of composite hydrogels, which showed great potential for application in health management and human–machine interfaces [204]. The functions of soft tissue protection, muscle contraction forces transmission during movement, ion maintenance, extracellular matrix (ECM), blood flow, and pH regulation are all performed. Bone is a highly specialized and vascular connective tissue. Bone may rebuild and mend itself after sustaining a slight damage. However, bone can no longer heal itself when abnormalities are bigger, as in the case of pathological fractures or traumatic or primary tumor excision [79].

The solubility and additional biomolecule functionalization capacity of MWCNT were improved by the biopolymer. Hevia et al. functionalized CNTs with targeted proteins to decrease nonspecific corona formation in biological media. Employing bovine serum proteins and an adsorption approach, they found remarkable increases in CNT mass and diameter, reflecting modified biological behaviors. The research indicates that the prefunctionalization of CNTs with targeted biomolecules can enhance their biocompatibility through the reduction of unwanted protein corona formation [205]. Vasconcelos et al. developed a 3D‐printed scaffold featuring a hexagonal honeycomb structure composed of a combination of CNTs, bioglass, and polylactic acid (PLA). This composite exhibited a high compressive strength of 0.56 GPa, along with extensive pore sizes and internal spacing. The scaffold effectively supported vascularization, cell proliferation, and differentiation, demonstrating excellent cell viability and thermal stability, as well as enhanced proliferation after 72 h [206]. Researchers studied the influence of the material on MC_3_T_3_‐E1 stem cells as well as the mechanical properties, surface hydrophilicity, and microstructures of the composite. Results showed that the addition of MWCNTs enhanced mechanical performance as such that the achieved mechanical values were on or even above those of human cortical bone. More importantly, studies using cell cultures demonstrated that MWCNTs/PEEK, and calcium polyphosphate greatly improved the initial adhesion, survival, and osteogenic differentiation of MC_3_T_3_‐E1 cells. In a related study, CNTs and powdered polymethyl methacrylate (PMMA) were used to make new bone. In vitro cultures of rat bone marrow mesenchymal stem cells (rBMSCs) on PMMA specimens with different MWCNT loading levels revealed that the addition of MWCNT improved cell adhesion and proliferation [207]. To help promote spinal cord repair and regeneration, Wang and colleagues developed the multifunctional poly‐citrate‐based NC hydrogel (PMEAC) scaffold, which has mechanical and electrical properties comparable to the spinal cord. PMEAC scaffolds were produced by simply self‐crosslinking poly(citrate‐maleic)‐polylysine (PME) and MWCNTs. The biomimetic mechanical modulus (*G*′ = 291.69 Pa) and electroconductivity (0.15 S/m) of PMEAC scaffolds containing spinal cord tissue were especially noteworthy, as were their injectable, tissue‐adhesive, broad‐spectrum antibacterial, and UV‐shielding properties. PMEAC scaffolds also show good cytocompatibility, hemocompatibility, and biodegradability. PMEAC scaffolds have been demonstrated to significantly improve locomotor recovery (BBB score 6–7), reduce inflammation, encourage remyelination, and support axon regeneration following spinal cord damage in vivo [208]. A thorough analysis of CNTs in the biomedical field was carried out by Murjani et al., with an emphasis on applications, preparation techniques, and functionalization strategies. Their study examined the application of CNTs in tissue engineering, cancer detection, and drug delivery [209]. Raphey et al. investigated CNTs as tools for tissue engineering, cancer detection, and the delivery of medications, genes, and vaccinations [210]. Abubakar et al. focused on the synthesis of CNT/natural rubber, discussing issues and uses outside of biomedicine, such as coatings, fire‐warning sensors, and acid storage tanks [211].

Although CNTs have shown promise as a promising tool in biomedical and diagnostics such as drug delivery, bioimaging, tissue engineering, and biosensing, there is an inadequate resolution of long‐term biocompatibility and toxicity. Recent reports have shown that cytotoxicity caused by CNTs is highly correlated with such physicochemical parameters as length, diameter, surface functionalization, aggregation state, and the presence of residual metal catalysts due to the synthesis processes [212, 213]. Long‐term in vivo exposure has been linked to possible risks associated with biodistribution, bioaccumulation of important organs, inflammatory and immunological reactions, and potential genotoxic effects [214, 215]. Whereas surface functionalization and purification approaches have been demonstrated to reduce acute toxicity and enhance dispersion, recent reviews highlight that there are still no standardized protocols of assessing long‐term toxicity as well as clinically relevant exposure models [216, 217]. These unresolved difficulties underscore the need for safer regulation and in vivo research, which can be accomplished through harmonized safety assessment methods, before CNT‐based biomedical technologies are reliably translated into clinical applications.

### 5.7 Environmental and Sensor Applications

Both CNTs and CNTCs have developed into multipurpose materials with potential applications in environmental monitoring and sensor technologies. By showing the effectiveness of CNCs, like SnO/CNT, in photocatalysis for the destruction of organic pollutants, recent work by Baroot et al. has emphasized the potential for environmental remediation [218]. In addition, through the study of the adsorption potential of CNTs and carbon soot in eliminating dyes from wastewater, George et al. published in 2024 illustrated the effectiveness of the materials in environmental remediation [219]. Furthermore, Inacio et al. research on the production of CNTs from LPG for sensor application emphasized its potential indetecting environmental pollutants [220]. Bajaj et al. developed an ammonia (NH_3_) sensor using cotton yarn and SWCNTs with two designs: Au/CNT/Au and all‐CNT (conducting, sensing, and conducting). In a chemi‐resistor configuration, they measured changes in resistance when exposed to NH_3_. The sensors demonstrated exceptional mechanical stability, uniformity, and reproducibility, making them promising candidates for low‐cost, textile‐based sensing applications [221]. Sunn et al. created a compact, CMOS‐compatible multimode gas sensor by integrating a CNT‐based chemiresistor with a MEMS bulk acoustic wave (BAW) resonator. This design enhances sensitivity, desorption kinetics, and manufacturing consistency through acoustic stimulation and BAW monitoring. In a prototype gas chromatography (GC) system, the sensor exhibited an improved dynamic range, low power consumption, and compatibility for portable gas detection applications [222].

While CNT‐based sensors are isotropic, conventional sensors have the disadvantage of being anisotropic. It is shown that CNT nanosensors have a higher order of magnitude of sensitivity than conventional solid‐state sensors [223]. CNT sensors have a larger surface area, are more compact, and have comparatively higher electrical and thermal conductivity than conventional sensors [224].

### 5.8 Other Applications

CNTs have an important role to play in creating next‐generation composite materials by being integrated into metal, ceramic, or polymer matrices to improve mechanical performance. Composites reinforced with CNTs how enhanced tensile strength, stiffness, and toughness and thus have applications in high‐performance uses in the automotive, aerospace, and building industries. Fabrication includes CNT purification and sonication, dispersion into the host matrix, mechanical blending, and controlled curing. Asidefrom structural uses, CNTs have also demonstrated significant potential for X‐ray production based on their efficiency in emitting electrons. By positioning a CNT cathode toward a metal‐coated anode, compact and energy‐conserving sources of X‐rays are attainable. This development opens doors to portable, low‐power X‐ray equipment that can be applied to medical diagnosis, industrial applications, and scientific exploration [225].

CNTs and their composites hold great potential for applications in water purification, notably in the manufacturing of filtration membranes. Utilizing the distinct nanoscale structure of CNTs, these filtration membranes effectively filter out contaminants and pollutants from water to potentially provide a clean water supply. These filtration membranes effectively remove diverse contaminants using the superior traits of CNTs, such as their mechanical strength and high surface area. They are thus significant instruments for resolving water quality problems [226, 227]. Li et al. employed LDH and CNTs to create a novel composite membrane for water filtration. Characterization indicated increased mass transfer channels that resulted in a gain in permeance of nearly 50%. Performance during separation was enhanced by 36% due to the optimization of fabrication conditions. The resultant membrane held stability for 100 h and exhibited 98% retention, indicating potential for use in water filtration applications [228, 229]. The special electronic characteristics of CNTs and their composites make them useful as diodes. Through the manipulation of CNTs structure and composition, scientists have created diode devices that can rectify electrical currents, allowing current to flow in one way while blocking it in another. Numerous electronic applications, such as ICs, optoelectronics, and sensor devices, have demonstrated potential for these CNT‐based diodes [230, 231]. The need for high‐performance resistive sensors with multipurpose monitoring capabilities is being driven by wearable electronics. A wearable 3D porous polyurethane (PU) sponge sensor coated with CNTs and MXene composites is presented by Wen et al. This sensor's ultrasonic dip‐coating assembly and robust conductive network enable it to measure a broad range of strains, from compressive to stretching. Wearable technology, healthcare, and human–machine interface can all benefit from the MXene/CNTs@PU sensor's exceptional electrical response and stability [232].

Chen et al. investigate the thermal conductivity of composite films composed of SWCNTs and rGOs. Unlike graphene or virgin CNTs, these composite films exhibit a distinct temperature‐dependent pattern that progressively increases in‐plane thermal conductivities at rising temperatures. This attribute enables applications like flexible thermal diodes. The two studies show the potential and adaptability of CNT‐based materials in developing technological fields [233].

### 5.9 Catalysis

#### 5.9.1 Catalytic Supports

**FIGURE 15:**
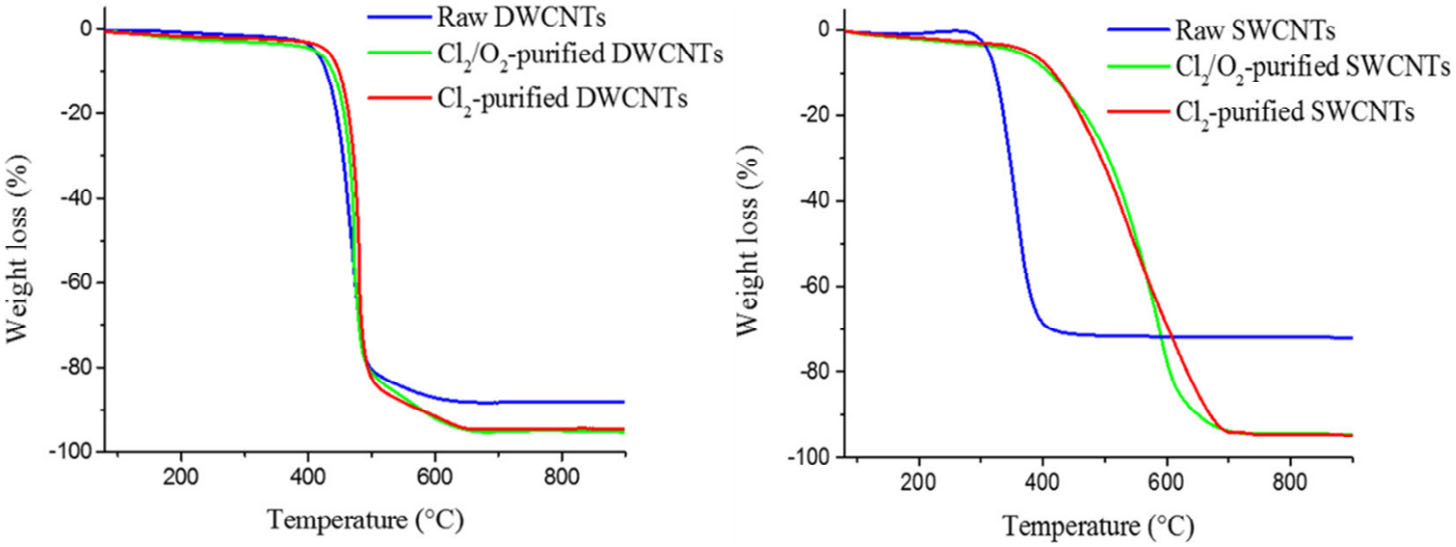
TGA curves for the raw and selected CNTs treated with Cl_2_ and Cl_2_/O_2_. Adapted from [236].

Catalytic supports because of their exceptional mechanical and thermal stability, high surface area, and tunable electronic characteristics, CNTs and their composites have attracted a lot of interest in the field of catalysis. These materials have been employed as cocatalysts, catalyst supports, and even catalysts in an array of reactions. When compared to conventional catalysts, CNT‐based catalysts have shown improved catalytic activity, selectivity, and stability in applications such as chemical synthesis, CO_2_ reduction, hydrogen evolution reaction (HER) [234], and oxygen reduction reaction (ORR) [235]. In a CNTs study by Berrada et al., transition metal‐based impurities accounted for around 10% of the sample weight loss. TGA profiles for DWCNT samples before and after purification show comparable combustion temperatures, while raw SWCNTs burned off at around 350°C, 50°C higher than purified DWCNTs. Figure 15 shows the characteristic behavior of CNT samples post‐Cl_2_ and Cl_2_/O_2_ treatments [236].

#### 5.9.2 Welding

Due to their enhanced properties and performance, CNTs and their composites have found numerous uses in the welding industry. One noteworthy use is in nanospot welding, where CNTs are applied to produce strong and reasonably priced patterns. A solder flow rate of 120  ag/s may be attained by filling CNTs with copper solder and applying a potential, demonstrating the effectiveness of CNT‐based welding systems. Additionally, CNTs are incorporated into magnesium composites for filler rods used in welding processes [231, 237].

#### 5.9.3 Lamps

Recent researchers have focusedonincorporating CNTs and CNCsinto lighting into lighting components to enhance performance and efficiency. Zhang et al. created improved electrical conductivity and thermal stability of CNT‐based composites as electrodes and heat dissipation in LED lamps, leading to greater life times and luminous efficiency [238]. Bhadra et al. also discussed the physical and chemical properties of different carbon structures, such as CNTs, carbon black, graphite, graphene, and fullerenes. These substances are used either directly or as fillers in composites made of polymers. The study explores the use of carbon fillers in polymer composites for a variety of uses, such as EMI shielding, microelectronics, OLEDs, sensors, and batteries [239]. Overall, new studies in this field show how CNTs and their composites can increase the durability, performance, and efficiency of lamps, opening the door to produce cutting‐edge lighting systems that are more functional and sustainable.

## 6 Conclusion and Future Prospects

New advances in the synthesis, functionalization, and processing of CNTs and their composites (CNTCs) have proved their gigantic potential in a variety of fields, most notably in energy storage, biomedical engineering, and environmental cleaning. Yet challenges persist most notably in the adjustment of CNTs to applications. For example, in CVD synthesis, the diameter of CNTs relies heavily on the size of the catalyst. Hence, the manufacturing of well‐controlled catalyst particles is crucial to provide structural homogeneity. Similarly, the high production cost of CNTs, based on the use of costly carbon precursors and elaborate purification protocols, remains the bottleneck for their commercialization on a large scale. Evolutionary needs from the microelectronics world underline the potential of SWCNT composites for facilitating nanoscale integration of circuits and substituting classical X‐ray filaments with more wear‐resistant, lightweight options. CNTs are also set to transform energy conversion, storage devices, and next‐generation biomedical devices. To observe their full potential, upcoming studies need to centre on scalable, low‐cost, and contamination‐free synthesis processes. Essential parameters, such as catalyst choice, gas condition, thermal control, and postsynthesis treatment, require systematic optimization. It is also of fundamental importance to assess the properties of CNTCs before and following functionalization to understand better the impact of chemical alterations. Additionally, CNTs and their composites hold great potential for enabling sustainable, high‐performance solutions in multidisciplinary areas. Further innovation in synthesis and processing will play a key role in advancing CNT‐based technologies from the laboratory to the marketplace.

## Conflicts of Interest

The authors declare no conflicts of interest.

## Data Availability

The data that support the findings of this study are available from the corresponding author upon reasonable request.
